# Epstein-Barr Virus Seropositivity, Immune Dysregulation, and Mortality in Pediatric Sepsis

**DOI:** 10.1001/jamanetworkopen.2025.27487

**Published:** 2025-08-19

**Authors:** Aditya Sriram, Kate F. Kernan, Yidi Qin, Zachary Aldewereld, Andrew H. Walton, Stephanie Cabler, Gregory Storch, Valerie Cheynet, Karen Brengel-Pesce, Scott Canna, Joseph A. Carcillo, Robert A. Berg, Kathy L. Meert, Murray Pollack, Mark Hall, Kit Newth, Rick Harrison, Tom Shanley, Kenneth E. Remy, Hyun Jung Park

**Affiliations:** 1 Department of Human Genetics, Graduate School of Public Health, University of Pittsburgh, Pittsburgh, Pennsylvania; 2 Department of Pediatrics and Critical Care Medicine, Washington University in St Louis, St Louis, Missouri; 3 Department of Pediatrics, Washington University in St Louis, St Louis, Missouri; 4 bioMerieux, Marcy-l'Étoile, France; 5 Department of Pediatrics, Perelman School of Medicine, University of Pennsylvania, Philadelphia; 6 Department of Anesthesiology, University of Pennsylvania, Philadelphia; 7 Department of Pediatrics, Central Michigan University, Detroit; 8 Department of Pediatrics, George Washington University, Washington, DC; 9 Department of Pediatrics, The Ohio State University, Columbus; 10 Department of Pediatrics, University of Southern California, Los Angeles; 11 Department of Pediatrics, University of California, Los Angeles; 12 Department of Pediatrics, University of Michigan, Ann Arbor; 13 Department of Pediatrics, Case Western Reserve University, Cleveland, Ohio

## Abstract

**Importance:**

Epstein-Barr virus (EBV) seropositivity is associated with chronic immune dysregulation conditions, including multiple sclerosis, systemic lupus erythematosus, post–COVID-19 condition, and multiple cancers. Sepsis is an acute immune dysregulation condition attributed to 1 of 5 global deaths.

**Objective:**

To assess causal associations among EBV seropositivity, immune dysregulation, and mortality in children with sepsis.

**Design, Setting, and Participants:**

This cohort study analyzed 320 children with sepsis in the 9-center Eunice Kennedy Shriver National Institutes of Child Health and Development Collaborative Pediatric Critical Care Research Network Phenotyping Pediatric Sepsis-Induced Multiple Organ Failure (PHENOMS) study who had not previously received intravenous immune globulin. Blood samples and clinical data were collected from January 1, 2015, to December 31, 2018, and assayed from January 1, 2019, to December 31, 2022. Causal algorithms were modeled in directed acyclic graphs and subsequent sensitivity and mediation analyses applied with further confirmation by structural equation modeling. Data analysis was performed from May 2022 to January 2025.

**Intervention:**

Blood sample collected at 24 to 48 hours of sepsis.

**Main Outcomes and Measures:**

Circulating biomarkers of inflammation (C-reactive protein, ferritin, and 32 cytokines), immune depression (ex vivo tumor necrosis factor response to endotoxin < 200 pg/mL), thrombotic microangiopathy (ADAMTS13 activity <57%), and EBV seropositivity (viral capsid IgG) were measured. Causal inference analysis identified causal associations between EBV seropositivity, immune dysregulation biomarkers, macrophage activation syndrome, and death.

**Results:**

Of the 320 children (median [IQR] age, 6 [1-12] years; 172 [53.8%] male), 150 (46.9%) were previously healthy, and 72 (22.5%) had immunocompromise at admission. A total of 172 (53.8%) had causal associations with death directly and through the mediators hyperferritinemia and macrophage activation syndrome (MAS) and also had direct causal associations with increased C-reactive protein, ferritin, and interleukin 18 binding protein, which in turn had direct causal associations with decreased ADAMTS 13 activity and decreased whole blood ex vivo tumor necrosis factor response to endotoxin. Mediation analysis found that EBV seropositivity was associated with mortality (estimate [SE], 1.86 [0.55]; *P* < .001). With both EBV seropositivity and ferritin included in the model, the effect of EBV seropositivity on death remained (estimate [SE], 1.52 [0.57]; *P* = .007), as did the ferritin effect (estimate [SE], 0.50 [0.15]; *P* = .001). EBV seropositivity remained significantly associated with death even after adjustment for MAS (estimate [SE], 1.78 [0.56]; *P* = .001).

**Conclusions and Relevance:**

In this cohort study of pediatric sepsis, EBV seropositivity was associated with immune dysregulation and mortality. Further study is warranted to address the possibility that latent EBV infection immune reprogramming poses an important public health problem that contributes to not only chronic disorders of immune dysregulation but also acute disorders of immune dysregulation, such as sepsis.

## Key Points

**Question:**

Is Epstein-Barr virus (EBV) seropositivity associated with immune dysregulation and death during pediatric sepsis?

**Findings:**

In this cohort study of 320 children with sepsis, EBV seropositive status was associated with death directly and through the mediators hyperferritinemia and macrophage activation syndrome.

**Meaning:**

These results suggest that latent EBV infection may be an important public health problem that contributes to chronic disorders of immune dysregulation and also acute disorders of immune dysregulation, such as pediatric sepsis.

## Teaser

This cohort study of children with sepsis assesses whether Epstein-Barr virus seropositivity is associated with the development of acute immune dysregulation reflected by biomarkers of hyperinflammation, immune depression, and thrombotic microangiopathy.

## Introduction

Latent Epstein-Barr virus (EBV) infection reflected by EBV IgG seropositivity is ubiquitous, affecting more than 90% of adults and 40% to 90% of children, depending on global location.^1,2,3,4,5^ EBV seropositivity is associated with development of chronic immune dysregulation–related conditions, including multiple sclerosis, systemic lupus erythematosus, post–COVID-19 condition, a variety of lymphoproliferative disorders (including B-cell lymphoma), and several other cancers (eg, nasoepithelial cell carcinoma).^6,7,8,9,10,11,12,13,14,15,16,17,18,19,20,21,22,23,24,25^ A developing theory holds that latent EBV infection causes host epithelial and immune cell reprogramming that permits virus escape and promotes immune dysregulation in these disorders.^26,27,28,29,30,31,32,33^

Sepsis is defined by infection and acute immune dysregulation, leading to organ failure. A recent global autopsy audit found 1 of 5 deaths was attributable to this condition.^34^ A previous study^35^ found that increased mortality was associated with EBV capsid antigen IgG seropositive status but not EBV DNAemia polymerase chain reaction (PCR) positivity, supporting EBV latency reprogramming rather than active EBV infection as a potential contributor to adverse outcome. In this study, we tested the hypothesis that EBV seropositivity is associated with development of acute immune dysregulation reflected by biomarkers of hyperinflammation, immune depression, and thrombotic microangiopathy in this same pediatric sepsis cohort. Because EBV capsid antigen IgG antibodies can be transfused with IVIG, and to a lesser extent with blood transfusion,^36,37^ we excluded children with sepsis who previously received intravenous immunoglobulin (IVIG) therapy and performed analyses on the whole sepsis cohort and in the subset of children who did not receive prior blood product exposure in the pediatric intensive care unit.

## Methods

This cohort study was approved by the central institutional review board of the University of Utah.^38^ Written informed consent was obtained from 1 or more parents or guardians for each child. Written assent was garnered when the child was able. This report follows the Strengthening the Reporting of Observational Studies in Epidemiology (STROBE) reporting guideline for cohort studies.

### Protocol

Clinical data and biobanked samples available from children previously enrolled in this 9-center Eunice Kennedy Shriver National Institutes of Child Health and Development Collaborative Pediatric Critical Care Research Network Phenotyping Pediatric Sepsis-Induced Multiple Organ Failure (PHENOMS) study were analyzed.^38^ Patients were enrolled from January 1, 2015, to December 31, 2017. The details of the clinical protocol have been previously published.^38^ Children qualified for enrollment if they (1) were between the ages of 44 weeks’ gestation to 18 years, (2) were suspected of having infection and had 2 or more of 4 systemic inflammatory response criteria, (3) had 1 or more organ failures, and (4) had an indwelling arterial line or central venous catheter for blood drawing.

Clinical data were assessed daily until 28 days or discharge from the pediatric intensive care unit (PICU). These data included demographic variables (age, sex, race and ethnicity by parental report, previously healthy status, and postoperative status), Pediatric Risk of Mortality Score 3 to assess severity of illness at admission, and organ failures. Parent-reported data on race and ethnicity were attained according to National Institutes of Health requirements. Blood samples were obtained in the first week at 24 to 48 hours before any receipt of IVIG. C-reactive protein (CRP), ferritin, 32 cytokines, ADAMTS13 activity (<57% activity is a biomarker of thrombotic microangiopathy), whole blood ex vivo tumor necrosis factor (TNF) response to endotoxin (<200 pg/mL is a biomarker of immune depression), EBV DNAemia PCR, and EBV-specific viral capsid antigen (VCA) IgG were measured.^35,38,39,40^ Macrophage activation syndrome (MAS) was defined by a platelet count less than 100 × 10^3^/μL (to convert to ×10^9^/L, multiply by 1), international normalized ratio greater than 1.5, bilirubin level greater than 1.0 mg/dL (to convert to micromoles per liter, multiply by 17.104), aspartate aminotransferase level greater than 100 IU/L (to convert to microkatals per liter, multiply by 0.0167), and alanine aminotransferase level of 100 IU/L (to convert to microkatals per liter, multiply by 0.0167).^38^ Organ failures were defined by modified Proulx criteria and death by hospital death.^38^ Patients were defined as being EBV seropositive based on detection of EBV-specific VCA IgG. Presumed EBV latent infection without reactivation was defined by EBV VCA IgG–positive, EBV PCR–negative status. No EBV infection was defined by EBV VCA–negative, EBV PCR–negative status.^35^

### Causal Discovery Algorithm Generation

In the causal discovery algorithms derived in our analysis, we used prior knowledge and a priori restricted the direction of the potential networks by setting the rule that EBV VCA antibody positivity preceded death because patients died after the time of sampling for EBV VCA antibody. We further used prior knowledge obtained from our univariate and multivariate analyses of admission characteristics that were associated with EBV VCA seropositivity to restrict the set of potential networks. In our primary causal inference analysis, we excluded these identified admission confounders. Then to assess whether this approach allowed residual exposure–outcome or mediator-outcome confounding to affect observed associations among included variables, we performed a second causal inference analysis including these confounders for both the whole population and the population that had not received transfusion. Having found similar results, we performed subsequent analyses in other subsets of interest (age >18 months vs ≤18 months, EBV latency vs no EBV) with the admission confounders excluded.

### Identifying Potential Causal Associations Using CausalMGM and DAG

We identified potential causal associations between 32 cytokines and functional biomarkers collected from 320 children admitted to the PICU for sepsis using a mixed graphical model (MGM) algorithm called causalMGM to initiate the inference of causal associations.^41^ This analysis was performed both with and without the set of confounders identified from the univariate regression. CausalMGM is an extension of Lee and Hastie’s MGM that estimates a Markov random field over a set of random variables. The Markov random field for the graphical node representing a random variable is a set of all the other random variables the random variable is conditionally dependent on. Due to the conditional dependence, the Markov random field of variable T contains the minimal set of variables that are necessary to estimate T, including only the direct causes (parents), direct effects (children), and direct causes of the direct effects (spouses) of T. With the Markov random field as a focused set of variables to examine for causal associations, a graphical model of the Markov random field enables users to examine their data to identify the direct effects on a chosen variable of interest or to discover new associations between variable pairs. We conducted an initial set of causal inference analyses for the full sample size (n = 320) and a second set using patients who did not receive blood product transfusions (n = 218), identifying causal associations using causalMGM plus directed acrylic graph (DAG) analyses.

### Loop-Erased Random Walks as a Pathway Analysis Framework

To determine the causal directions in the graphical model (undirected graph) from a potential source of sepsis to death, we used random walks (eFigure 2 in Supplement 1). Broadly, within the context of network biology, a random walk is a process where a sequence of steps is taken randomly from one node to another, going through the network to explore its structure and any potential association between features in the network.^42^ Recently, random walks have been used to determine causal directions in biological networks.^43,44^ For example, Tu et al^45^ used a heuristic random walk in an integrated network to find regulatory modules in gene expression data, identifying the most likely paths from quantitative trait loci to a candidate gene. Often in network graphs, random walks inherently have self-looping, which refers to walks that begin and end at the same node. Additionally, to explore as much of the network space as possible without informational redundancies due to self-looping, we implemented loop erasure.

To identify potentially causal pathways from EBV seropositivity to death, we implemented a loop-erased random walk (LERW) algorithm on our set of vertices and edges identified by causalMGM. In a LERW construct, any time a loop is formed the loop is immediately erased.^46^ To initialize paths for our random walks, we start the random walk at vertex *s*, setting the initial path *P* = [*s*] and label our *sink* node specifying where walks should end. In our setup, *s* = EBV seropositivity phenotype and our sink node is death. We use 500 random walks for all analyses. The network graph is weighted according to causal association effect sizes identified by the causalMGM algorithm. Larger effect size values indicate stronger causal associations.

### Subnetwork Identification and Network Graph Drawings

The LERW analysis generated a list of all paths from EBV seropositivity to death. To highlight the most prominent nodes, we normalized all nodes in each pathway by the total length and aggregated them. Then, we used the mean distance to death across all the random walks as a metric to highlight which nodes were causally the closest to death. Nodes that did not get visited at least 25 times (<5%) were not included as part of the resulting subnetworks. We used Cytoscape, version 3.9.1 (Cytoscape Consortium) for the visualization of our feature phenotype causal association results.^47^ Arrow widths in the network graphs are proportional to the derived effect sizes from the causalMGM algorithm.

### Sensitivity and Mediation Analysis

To assess any potential effect resulting from unmeasured confounding on observed causal associations between exposures and the defined death phenotype, we performed sensitivity testing by running an E-value analysis for each identified exposure-outcome association for the whole cohort and the nontransfused group. Additionally, to assess mediation, we evaluated whether ferritin levels and MAS acted as mediators of the EBV seropositivity and death association using the Baron-Kenny mediation analysis framework^48^

### Structural Equation Modeling

We applied structural equation modeling to validate our causal discovery method findings of direct and indirect associations among EBV seropositivity, mediators, and mortality using the lavaan R Package (R Project for Statistical Computing).^49^ Models were run independently for the full patient cohort and the nontransfused group, with and without mediation paths, to assess both total and mediated effects. Systems equation modeling allows for simultaneous estimation of causal pathways while accounting for latent structure and intervariable correlations. The eMethods in Supplement 1 provide expansion of the causal algorithm, sensitivity and mediation, and structural equation modeling analyses presented in the text.

### Statistical Analysis

eFigure 1 in Supplement 1 provides a flow chart of our study analyses. We compared admission characteristics, outcomes, 32 cytokines, and functional biomarkers for patients across EBV serologic categories. We performed univariate regression analysis to identify features associated with EBV seropositivity and then conducted multivariable regression to validate EBV seropositivity’s association with mortality after accounting for these confounders from the initial univariate regression. We present continuous variables as median (IQR) or mean (SD) and categorical variables as percentages. To compare patients visually, we used heatmaps with hierarchical clustering. To compare patients statistically, we used Kruskal-Wallis tests for continuous data and the χ^2^ test for categorical data. Fisher exact tests were applied for groups containing fewer than 5 individuals. The threshold for statistical significance was a 2-sided *P* < .05 after adjustment for multiple testing. Holm-Bonferroni correction was applied to correct for multiple testing. All analyses were performed with R, version 3.6.2 (R Project for Statistical Computing).

## Results

Among 401 children enrolled with sepsis, 320 children who had not previously received IVIG in the PICU (median [IQR] age, 6 [1-12] years; 172 [53.8%] male and 148 [46.2%] female; 14 [4.4 %] Asian, 64 [20.0%] Black, 219 [68.4%] White, 25 [11.4%] unknown race) were studied. Of these 320 children 150 (46.9%) were previously healthy and 72 (22.5%) were immunocompromised (Table). Of these 320 children, 172 (53.8%) were EBV seropositive, with 26 deaths (15.1%), and 148 (46.2%) were EBV seronegative, with 4 deaths (2.7%). A total of 218 (68.1%) did not receive earlier PICU blood transfusion before being assayed for EBV serologic status, of whom 95 (43.6%) were EBV seropositive, with 8 deaths (8.4%), and 123 (56.4%) were EBV seronegative, with 1 death (0.8%). EBV seropositivity was associated with older age, immunocompromised status, increased Pediatric Risk of Mortality Score 3 scores, more organ failures, and decreased viral infections at admission (Table). After adjustment for these admission confounders, EBV seropositivity remained associated with increased mortality in all 320 children (adjusted odds ratio [OR], 6.1; 95% CI, 2.1-17.9; *P* = .001) as well as in the subset of 218 children without earlier blood product exposure in the PICU (adjusted OR, 9.06; 95% CI, 1.51-71.6; *P* = .03).

**Table.** Admission Characteristics of Serologic Testing for All Patients and Patients Without Transfusions^a^

Admission characteristic	All patients (N = 320)		Patients without transfusion (n = 218)	
	Positive serologic test results (n = 172)	Negative serologic test results (n = 148)	Positive serologic test results (n = 95)	Negative serologic test results (n = 123)
Age, median (IQR), y	7 (2-13)	3 (1-9)	9 (3-13)	3 (1-10)
Sex				
Female	83 (48.3)	65 (43.9)	49 (51.6)	56 (45.5)
Male	89 (51.7)	83 (56.1)	46 (48.4)	67 (54.5)
Race				
Asian	7 (4.1)	7 (4.7)	3 (3.2)	6 (4.9)
Black	40 (23.3)	24 (16.2)	22 (23.2)	19 (15.4)
White	111 (64.5)	108 (73.0)	64 (617.4)	90 (73.2)
Unknown	14 (8.1)	9 (5.4)	6 (6.3)	8 (6.5)
Ethnicity				
Non-Hispanic	134 (77.9)	119 (80.4)	72 (75.8)	95 (77.2)
Hispanic	30 (17.4)	26 (17.6)	20 (21.0)	25 (20.3)
Unknown	8 (4.6)	3 (1.4)	3 (3.2)	3 (2.4)
Previous healthy	72 (41.9)	78 (52.7)	46 (48.4)	70 (56.9)
Immunocompromised^b^	56 (32.6)	16 (10.8)	21 (22.1)	12 (9.8)
PRISM-3 score, median (IQR)	9 (5-15)	7 (3-12)	7 (3-13)	6 (3-11)
OFI, median (IQR)^c^	2 (1-2)	1 (1-2)	2 (1-2)	1 (1-2)
Bacterial infection	67 (40.0)	46 (31.1)	34 (35.8)	35 (28.5)
Viral infection	31 (18.0)	58 (39.2)	17 (17.9)	49 (39.8)
Coinfection^d^	13 (7.6)	11 (7.4)	6 (6.3)	7 (5.7)
Fungal infection	1 (0.6)	1 (0.7)	1 (1.0)	0

Abbreviations: PRISM-3, Pediatric Risk of Mortality Index 3; OFI, Organ Failure Index.

^a^ The Kruskal-Wallis test was used for continuous variables; the χ^2^ test or the Fisher exact test (group sample size <10) was used for discrete variables.

^b^ Immunocompromised indicates cancer, transplantation, or use of immunosuppressant therapies.

^c^ OFI is an integer score reflecting the number of organ failures. OFI scores are 0 or 1 for cardiovascular, hepatic, hematologic, respiratory, neurologic, and kidney and summed for a total range of 0 to 6.

^d^ Coinfection indicates bacteria and virus infections.

**Figure 1.**
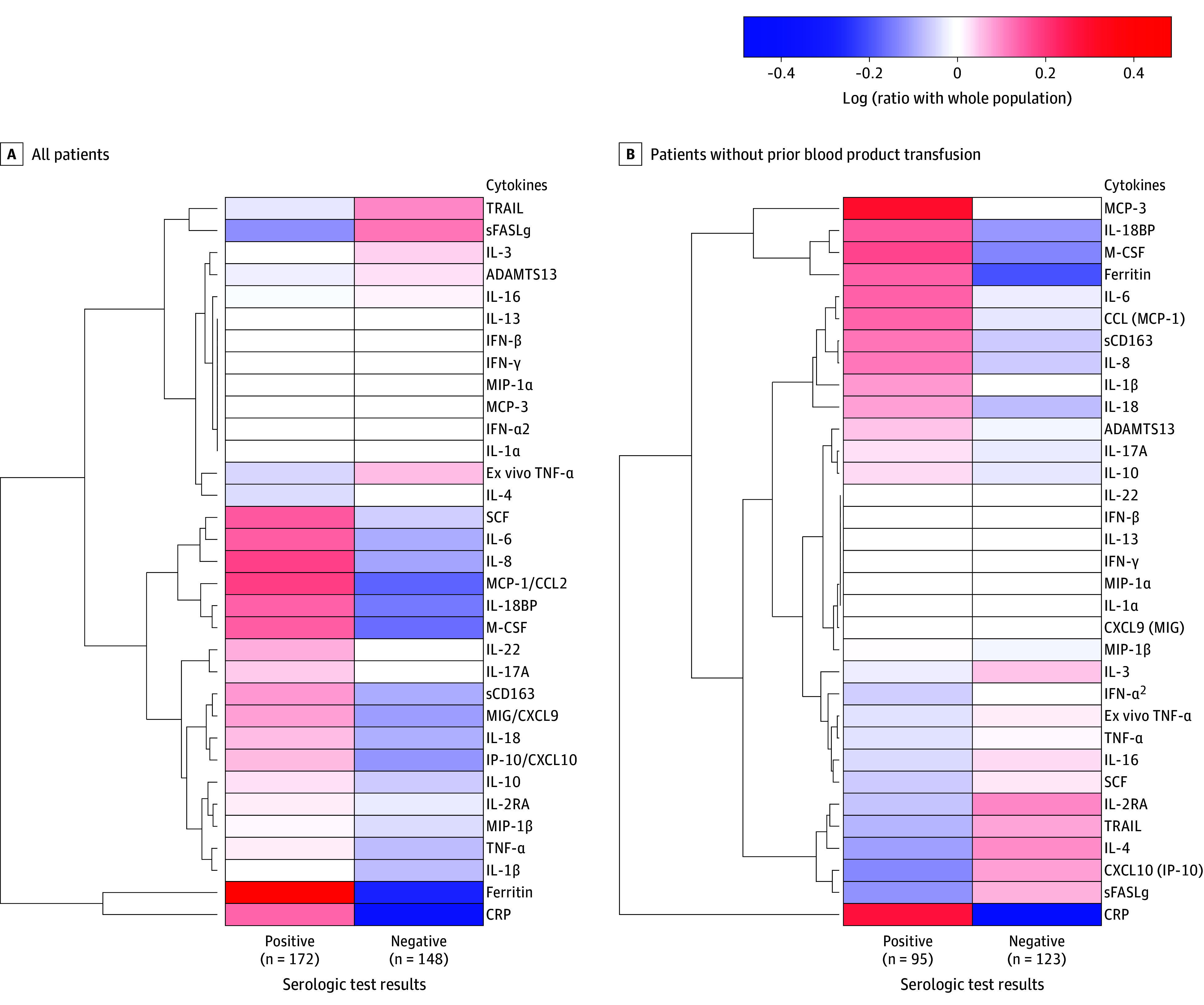
Cytokine Heatmap of Groups With Epstein-Barr Virus Positive and Negative Serologic Test Results The heatmaps in both panels show the log ratio of the median biomarker values for various markers of the host response and their hierarchical cluster associations. Red represents a greater median biomarker value for that group compared with the median for the entire study cohort, whereas blue represents a lower median biomarker value compared with the median for the entire study cohort. BP indicates binding protein; CRP, C-reactive protein; IFN, interferon; IL, interleukin; IP-10, interferon gamma-induced protein 10; MCP, monocyte chemoattractant protein; M-CSF, monocyte colony-stimulating factor; MIG, monokine induced by γ-interferon; MIP, macrophage inflammatory protein; sCD163, soluble CD163; SCF, stem cell factor; sFASLg, soluble FAS ligand; TNF, tumor necrosis factor.

Among the overall cohort of 320 children, the 172 EBV-seropositive children (53.8%) showed increased CRP, ferritin, soluble CD163, interleukin (IL) 18 binding protein (BP), CXCL9 (also known as monokine induced by γ-interferon), IL-6, IL-8, IL-10, CCL2 (also known as monocyte chemoattractant protein 1), macrophage inflammatory protein 1α, TNF, monocyte colony-stimulating factor, and stem cell factor levels, with decreased soluble FAS ligand and TNF-related apoptosis-inducing ligand levels compared with EBV-seronegative children (Figure 1A; eTable 1 in Supplement 1). Among the subset of 218 children who did not receive blood products in the PICU before measurement of their serologic status, the 95 EBV-seropositive children (43.6%) similarly showed increased CRP, ferritin, IL-18BP, and IL-8 levels with decreased TNF-related apoptosis-inducing ligand levels compared with EBV-seronegative children (Figure 1B; eTable 1 in Supplement 1).

**Figure 2.**
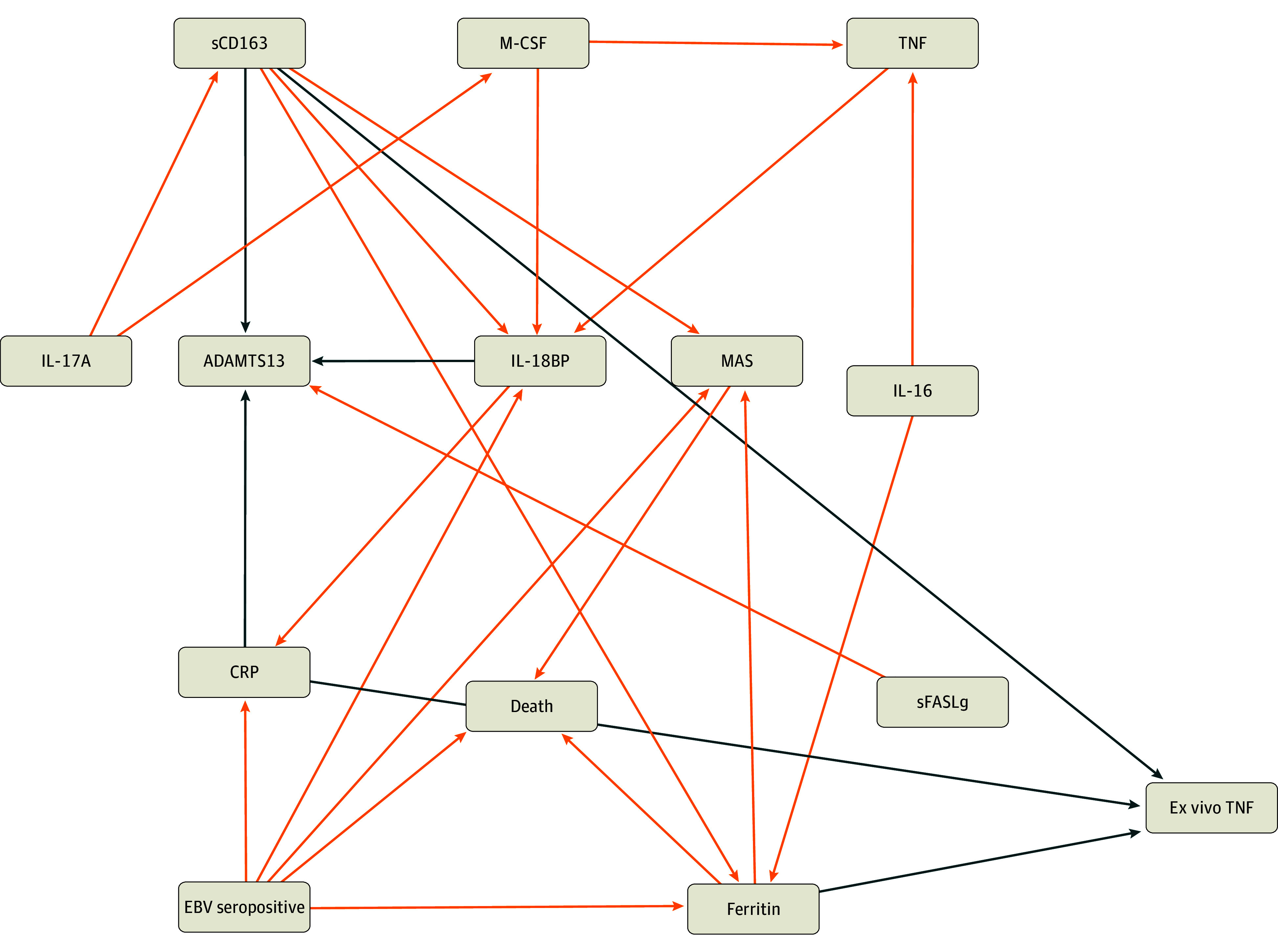
Abridged Causal Association Network for Epstein-Barr Virus (EBV) Seropositivity Phenotype in All 320 Patients Orange arrows represent positive effect sizes, and blue arrows represent negative effect sizes. See eFigure 3 in Supplement 1 for the full network. BP indicates binding protein; CRP, C-reactive protein; IL, interleukin; MAS, macrophage activation syndrome; M-CSF, monocyte colony-stimulating factor; sCD163, soluble CD163; SCF, stem cell factor; sFASLg, soluble FAS ligand; TNF, tumor necrosis factor.

**Figure 3.**
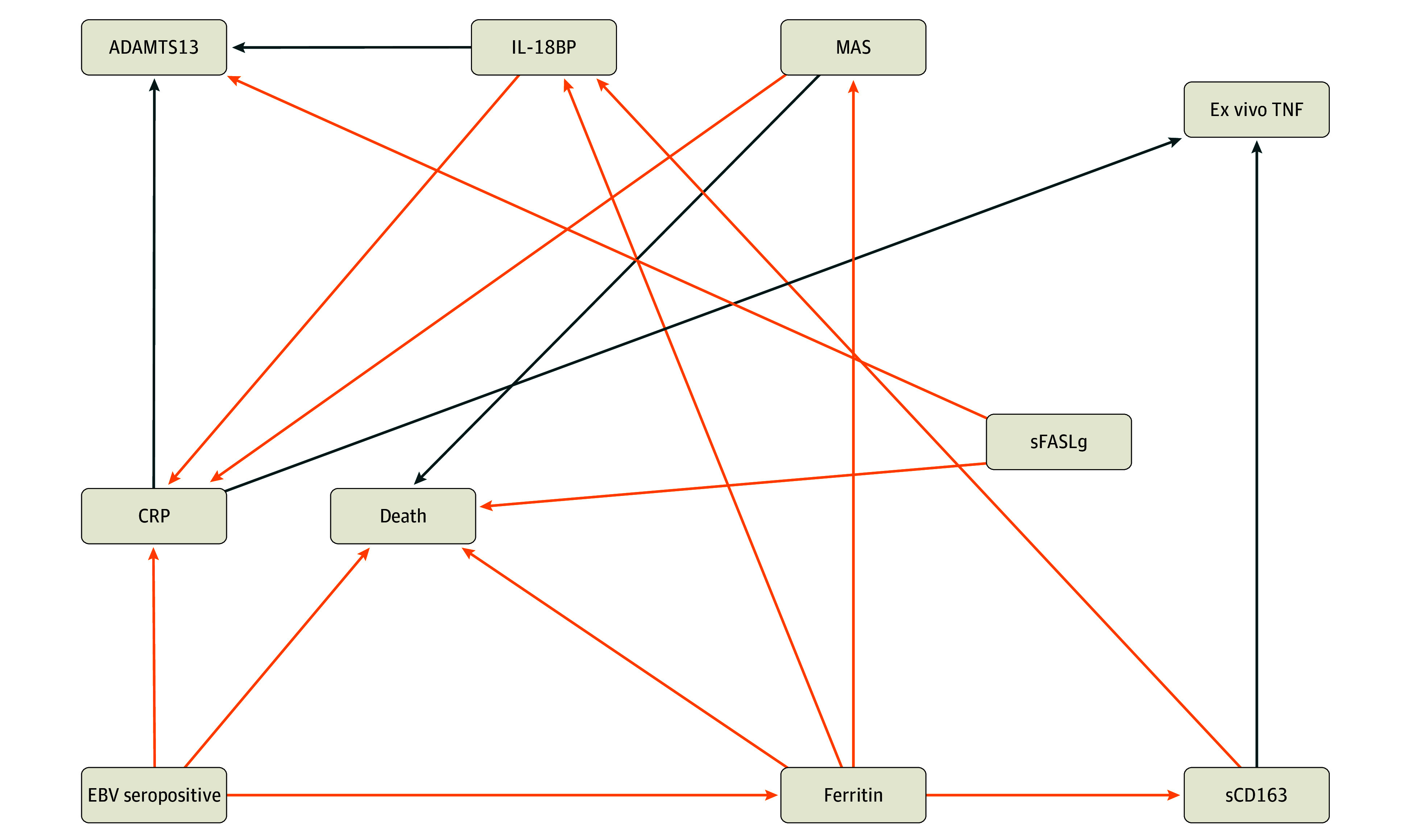
Abridged Causal Association Network for Epstein-Barr Virus (EBV) Seropositivity Phenotype in the 218 Patients Without Prior Transfusion Orange arrows represent positive effect sizes, and blue arrows represent negative effect sizes. See eFigure 4 in Supplement 1 for full network. BP indicates binding protein; CRP, C-reactive protein; IL, interleukin; MAS, macrophage activation syndrome; sCD163, soluble CD163; sFASLg, soluble FAS ligand; TNF, tumor necrosis factor.

**Figure 4.**
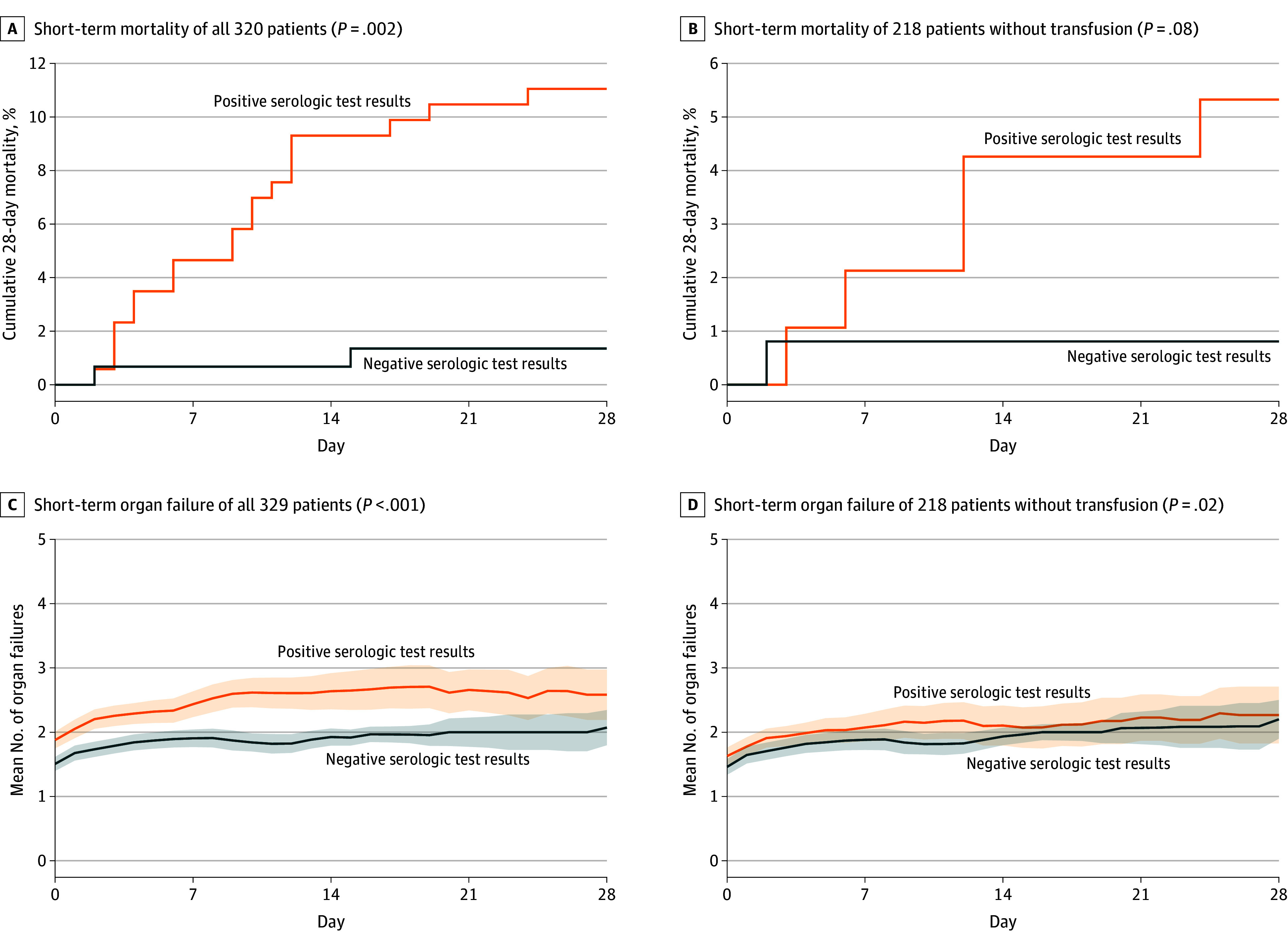
Outcome Curves for 28 Days in Groups With Positive and Negative Serologic Test Results The mean numbers of organ failures and 95% CIs (shaded areas) were calculated each day by nonnested observation; the Organ Failure Index is not carried forward at the time the patient leaves the pediatric intensive care unit alive or dead.

The directed acyclic graph (DAG) analyses demonstrate all MGM-derived causal association nodes with EBV-seropositive status (Figures 2, 3, and 4; eFigures 3 and 4 in Supplement 1). Among the entire 320-patient cohort, EBV seropositivity showed causal associations with death both directly and through the mediators hyperferritinemia and MAS (Figure 2; eFigure 3 in Supplement 1). Sensitivity analysis (eTable 2 in Supplement 1) showed that EBV seropositivity was associated with increased mortality in the full cohort (relative risk [RR], 5.07 [95% CI, 1.91-13.45]; E-value, 9.62) as was ferritin (RR, 3.73 [95% CI, 1.92-7.26]; E-value, 6.93), whereas MAS showed a weaker and less consistent association (RR, 2.80 [95% CI, 1.24-6.35]; E-value, 5.05), suggesting higher robustness of EBV and ferritin as factors associated with mortality. Mediation analysis (eTable 3 in Supplement 1) showed that EBV seropositivity was associated with mortality (estimate [SE], 1.86 [0.55]; *P* < .001). With both EBV seropositivity and ferritin included in the model, the effect of EBV seropositivity on death remained (estimate [SE], 1.52 [0.57]; *P* = .007), as did the ferritin effect (estimate [SE], 0.50 [0.15]; *P* = .001). EBV seropositivity remained significantly associated with death even after adjustment for MAS (estimate = 1.78 [0.56]; *P* = .001). Results were similar when comparing EBV seropositivity and mortality associations using structural equation modeling with and without mediation (eTable 4 in Supplement 1). EBV seropositivity further showed causal association with decreased whole blood ex vivo TNF response to endotoxin (biomarker of immune depression) through the mediators CRP and ferritin and with decreased ADAMTS 13 activity (biomarker of thrombotic microangiopathy) through the mediators CRP and IL-18BP.

The subset of 218 patients without prior blood exposure in the PICU similarly showed causal associations between EBV seropositivity and mortality directly and through the mediator hyperferritinemia (Figure 3; eFigure 4 in Supplement 1). Sensitivity analysis showed that EBV seropositivity was associated with increased mortality in the nontransfused group (RR, 7.46 [95% CI, 1.34-41.44]; E-value, 14.39) as was ferritin (RR, 3.28 [95% CI, 0.95-11.38]; E-value, 6.02), whereas MAS again showed a weaker and less consistent association (RR, 1.39 [95% CI, 0.09-21.92]; E-value, 2.14), reinforcing higher robustness of EBV and ferritin as factors associated with mortality that are less likely to be affected by unmeasured confounding (eTable 2 in Supplement 1). Results were similar when comparing EBV seropositivity and mortality associations using structural equation modeling with and without mediation (eTable 4 in Supplement 1). EBV seropositivity also showed causal associations with decreased whole blood ex vivo TNF response to endotoxin through the mediators CRP and ferritin (through the mediator soluble CD163) and with decreased ADAMTS 13 activity through the mediators CRP and ferritin (through the mediator IL-18BP).

DAG analyses similarly showed that EBV-seropositive status remained causally associated with immune dysregulation and death when the confounding admission characteristics were included as nodes (eFigures 5 and 6 in Supplement 1) and when limited to the children older than 18 months (eFigures 7 and 8 in Supplement 2). The immune dysregulation biomarker heatmap, causal inference, and mortality analyses findings also remained similar when limiting to comparison of the 139 children (49.6%) with latent EBV infection without presumed reactivation (EBV IgG positive and EBV PCR negative), with 21 deaths (15.1%) to the 141 children (50.4%) without EBV infection (EBV IgG negative and EBV PCR negative who had 4 deaths (2.8%) (OR, 6.10; 95% CI, 2.03-18.26; *z* statistic = 3.239; *P* = .001) (eTables 5-8 and eFigures 9-11 in Supplement 1).

## Discussion

To our knowledge, this is the first report to provide a causal inference analysis of the association of EBV seropositivity with mortality in pediatric sepsis. This analysis further identifies association of EBV seropositivity with immune dysregulation characterized by biomarkers of hyperinflammation (increased CRP, ferritin, and MAS), immune depression (decreased whole blood ex vivo TNF response to endotoxin), and thrombotic microangiopathy (decreased ADAMTS 13 activity). These observations were robust even in children who had not received prior blood transfusion in the PICU. These associations are consistent with the hypothesis that latent EBV infection contributes not only to chronic disorders of immune dysregulation but also possibly to acute disorders of immune dysregulation, such as sepsis, in children providing further rationale for EBV vaccine development initiatives.

The potential for therapeutically targeting the effects of latent EBV infection on immune metabolic reprogramming is under study.^50,51,52,53,54,55,56,57,58,59^ Weiss and colleagues^60^ reported that EBV latent infection–transformed lymphoblastoid cell lines exposed to endotoxin undergo oxidative phosphorylation uncoupling, which can be reversed with sodium butyrate treatment. In EBV latency–related nasoepithelial carcinoma and lymphoproliferative diseases, there is an emerging theory that LMP, an EBV latency protein, interacts with host AMP kinase to affect proliferation by uncoupling oxidative phosphorylation. Metformin restores AMP kinase activity, leading to reversal of proliferation with minimal toxic effects. A beneficial effect of metformin has similarly been reported in other latent EBV infection–attributable conditions, including multiple sclerosis, systemic lupus erythematosus, post-COVID-19 infection, and sepsis.^61,62,63,64,65^

### Limitations

This cohort study has important limitations. We presumed latent EBV infection based on VCA IgG antibody seropositivity because this laboratory test is used as a criterion standard diagnostic in clinical practice. We measured plasma VCA antibodies to EBV infection 24 to 48 hours after children had developed sepsis, introducing the potential for temporal ambiguity in causal inference. Ideally, future studies should aim to incorporate presepsis baseline serostatus to establish correct temporal sequence. Our study also lacks a control group of critically ill children without sepsis or children without critical illness. Hence, it is difficult to disentangle whether the observed associations are specific to the pathobiology of sepsis or generalizable to mechanisms of immune dysregulation in critical illness. Further causal discovery algorithms do not exclude unmeasured confounding or associations that would be observed had more complete data been available. Validation in independent, critically ill pediatric illness cohorts with and without sepsis is needed to overcome selection bias.

## Conclusions

In this cohort study of pediatric sepsis, the associations observed with EBV seropositivity support increasing literature suggesting that latent EBV infection is an important, underrecognized, and potentially remediable global public health problem. Further study is warranted to address the possibility that latent EBV infection immune reprogramming poses an important public health problem that contributes to not only chronic disorders of immune dysregulation but also acute disorders of immune dysregulation, such as sepsis.

## Notes

Supplement 1. **eFigure 1.** Study Flow Chart **eFigure 2.** Illustration of Random Walk Method **eMethods.** Extended DG Methods **eTable 1.** Biomarkers Measured With Serology Using All Patients and Patients Without Transfusion **eFigure 3.** Full Causal Association Network for EBV Seropositivity Phenotype in All Patients (n = 320) **eFigure 4.** Full Causal Association Network for EBV Seropositivity Phenotype in Patients Without Transfusion (n = 218) **eTable 2.** Sensitivity Analysis Risk Ratios and E-Values: EBV seropositivity, Ferritin, and MAS With Mortality **eTable 3.** Mediation Analysis: EBV Seropositivity, Ferritin, and MAS With Mortality **eTable 4.** Structural Equation Modeling Without and With Mediation **eFigure 5.** Full Causal Association Network for EBV Seropositivity Phenotype in All Patients (n = 320) Including Admission Confounders Increased Age, Organ Failures, Severity of Illness (PRISM Score), and Immunocompromise as Nodes **eFigure 6**. Full Causal Association Network for EBV Seropositivity Phenotype in Patients Without Transfusion (n = 218) Including Admission Confounders Increased Age, Organ Failures, Severity of Illness (PRISM Score), and Immunocompromise as Nodes **eFigure 7.** Full Causal Association Network for EBV Seropositivity Phenotype in All Patients Greater Than 18 Months Old (n = 237) **eFigure 8.** Full Causal Association Network for EBV Seropositivity Phenotype in Patients Greater Than 18 Months Old Without Transfusions (n = 167) **eTable 5.** Outcome Characteristics According to EBV Seropositivity Phenotype in All Patients and in Patients Without Transfusion **eTable 6**. Admission Characteristics of EBV Latency Without Presumed Reactivation (EBV VCA + / EBV PCR -) Compared to No EBV Infection (EBV VCA - / EBV PCR -) in All Patients and Patients Without Transfusio **eTable 7**. Biomarkers Measured by EBV Latency Without Presumed Reactivation (EBVCA + / EBV PCR -) Compared to No EBV Enfection (EBVVCA - / EBV PCR -) in All Patients and Patients Without Transfusion **eFigure 9.** Cytokine Heatmap of EBV Latent Without Presumed Reactivation (EBV VCA + / EBV PCR -) and No EBV (EBV VCA - / EBV PCR -) Groups **eFigure 10**. Abridged Causal Association Network for EBV Latency Without Presumed Reactivation (EBV VCA + / EBV PCR -) Compared to No EBV Infection (EBV VCA - / EBV PCR -) in All Patients **eFigure 11**. Outcome Curves Over 28 Days Among All Patients With EBV Latency Without Presumed Reactivation (EBV VCA + / EBV PCR -) and All Patients Without EBV Infection (EBV VCA - / EBV PCR -). **eTable 8.** Outcome Characteristics Comparing EBV Latency to No EBV Infection in All Patients and in Patients Without Transfusion

Supplement 2.
Data Sharing Statement
