# Supplementary material for: Epstein-Barr Virus Seropositivity, Immune Dysregulation, and Mortality in Pediatric Sepsis
Source: JAMA Netw Open. 2025 Aug 19;8(8):e2527487. doi: 10.1001/jamanetworkopen.2025.27487 (PMC12365707; doi:10.1001/jamanetworkopen.2025.27487)
Supplement: Supplement 1. — eFigure 1. Study Flow Chart eFigure 2. Illustration of Random Walk Method eMethods. Extended DG Methods eTable 1. Biomarkers Measured With Serology Using All Patients and Patients Without Transfusion eFigure 3. Full Causal Association Network for EBV Seropositivity Phenotype in All Patients (n = 320) eFigure 4. Full Causal Association Network for EBV Seropositivity Phenotype in Patients Without Transfusion (n = 218) eTable 2. Sensitivity Analysis Risk Ratios and E-Values: EBV seropositivity, Ferritin, and MAS With Mortality eTable 3. Mediation Analysis: EBV Seropositivity, Ferritin, and MAS With Mortality eTable 4. Structural Equation Modeling Without and With Mediation eFigure 5. Full Causal Association Network for EBV Seropositivity Phenotype in All Patients (n = 320) Including Admission Confounders Increased Age, Organ Failures, Severity of Illness (PRISM Score), and Immunocompromise as Nodes eFigure 6. Full Causal Association Network for EBV Seropositivity Phenotype in Patients Without Transfusion (n = 218) Including Admission Confounders Increased Age, Organ Failures, Severity of Illness (PRISM Score), and Immunocompromise as Nodes eFigure 7. Full Causal Association Network for EBV Seropositivity Phenotype in All Patients Greater Than 18 Months Old (n = 237) eFigure 8. Full Causal Association Network for EBV Seropositivity Phenotype in Patients Greater Than 18 Months Old Without Transfusions (n = 167) eTable 5. Outcome Characteristics According to EBV Seropositivity Phenotype in All Patients and in Patients Without Transfusion eTable 6. Admission Characteristics of EBV Latency Without Presumed Reactivation (EBV VCA + / EBV PCR -) Compared to No EBV Infection (EBV VCA - / EBV PCR -) in All Patients and Patients Without Transfusio eTable 7. Biomarkers Measured by EBV Latency Without Presumed Reactivation (EBVCA + / EBV PCR -) Compared to No EBV Enfection (EBVVCA - / EBV PCR -) in All Patients and Patients Without Transfusion eFigure 9. Cytokine Heatmap of EBV Lat [file jamanetwopen-e2527487-s001.pdf]

Supplemental Online Content

Sriram A, Kernan KF, Qin Y, et al. Epstein-Barr virus seropositivity, immune dysregulation, and mortality in pediatric sepsis. *JAMA Netw Open*. 2025;8(7):e2527487. doi:10.1001/jamanetworkopen.2025.27487

eFigure 1. Study Flow Chart
eFigure 2. Illustration of Random Walk Method
eMethods. Extended DG Methods
eTable 1. Biomarkers Measured With Serology Using All Patients and Patients Without Transfusion
eFigure 3. Full Causal Association Network for EBV Seropositivity Phenotype in All Patients (n = 320)
eFigure 4. Full Causal Association Network for EBV Seropositivity Phenotype in Patients Without Transfusion (n = 218)
eTable 2 Sensitivity Analysis Risk Ratios and E-Values: EBV seropositivity, Ferritin, and MAS With Mortality
eTable 3. Mediation Analysis: EBV Seropositivity, Ferritin, and MAS With Mortality
eTable 4. Structural Equation Modeling Without and With Mediation
eFigure 5. Full Causal Association Network for EBV Seropositivity Phenotype in All Patients (n = 320) Including Admission Confounders Increased Age, Organ Failures, Severity of Illness (PRISM Score), and Immunocompromise as Nodes
eFigure 6. Full Causal Association Network for EBV Seropositivity Phenotype in Patients Without Transfusion (n = 218) Including Admission Confounders Increased Age, Organ Failures, Severity of Illness (PRISM Score), and Immunocompromise as Nodes
eFigure 7. Full Causal Association Network for EBV Seropositivity Phenotype in All Patients Greater Than 18 Months Old (n = 237)
eFigure 8. Full Causal Association Network for EBV Seropositivity Phenotype in Patients Greater Than 18 Months Old Without Transfusions (n = 167)
eTable 5. Outcome Characteristics According to EBV Seropositivity Phenotype in All Patients and in Patients Without Transfusion
eTable 6. Admission Characteristics of EBV Latency Without Presumed Reactivation (EBV VCA + / EBV PCR -) Compared to No EBV Infection (EBV VCA - / EBV PCR -) in All Patients and Patients Without Transfusion
eTable 7. Biomarkers Measured by EBV Latency Without Presumed Reactivation (EBVCA + / EBV PCR -) Compared to No EBV Enfection (EBVVCA - / EBV PCR -) in All Patients and Patients Without Transfusion
eFigure 9. Cytokine Heatmap of EBV Latent Without Presumed Reactivation (EBV VCA + / EBV PCR -) and No EBV (EBV VCA - / EBV PCR -) Groups
eFigure 10. Abridged Causal Association Network for EBV Latency Without Presumed Reactivation (EBV VCA + / EBV PCR -) Compared to No EBV Infection (EBV VCA - / EBV PCR -) in All Patients
eFigure 11. Outcome Curves Over 28 Days Among All Patients With EBV Latency Without Presumed Reactivation (EBV VCA + / EBV PCR -) and All Patients Without EBV Infection (EBV VCA - / EBV PCR -)
eTable 8. Outcome Characteristics Comparing EBV Latency to No EBV Infection in All Patients and in Patients Without Transfusion

This supplemental material has been provided by the authors to give readers additional information about their work.

© 2025 Sriram A et al. *JAMA Network Open.*

eFigure 1 – Study Flow Chart

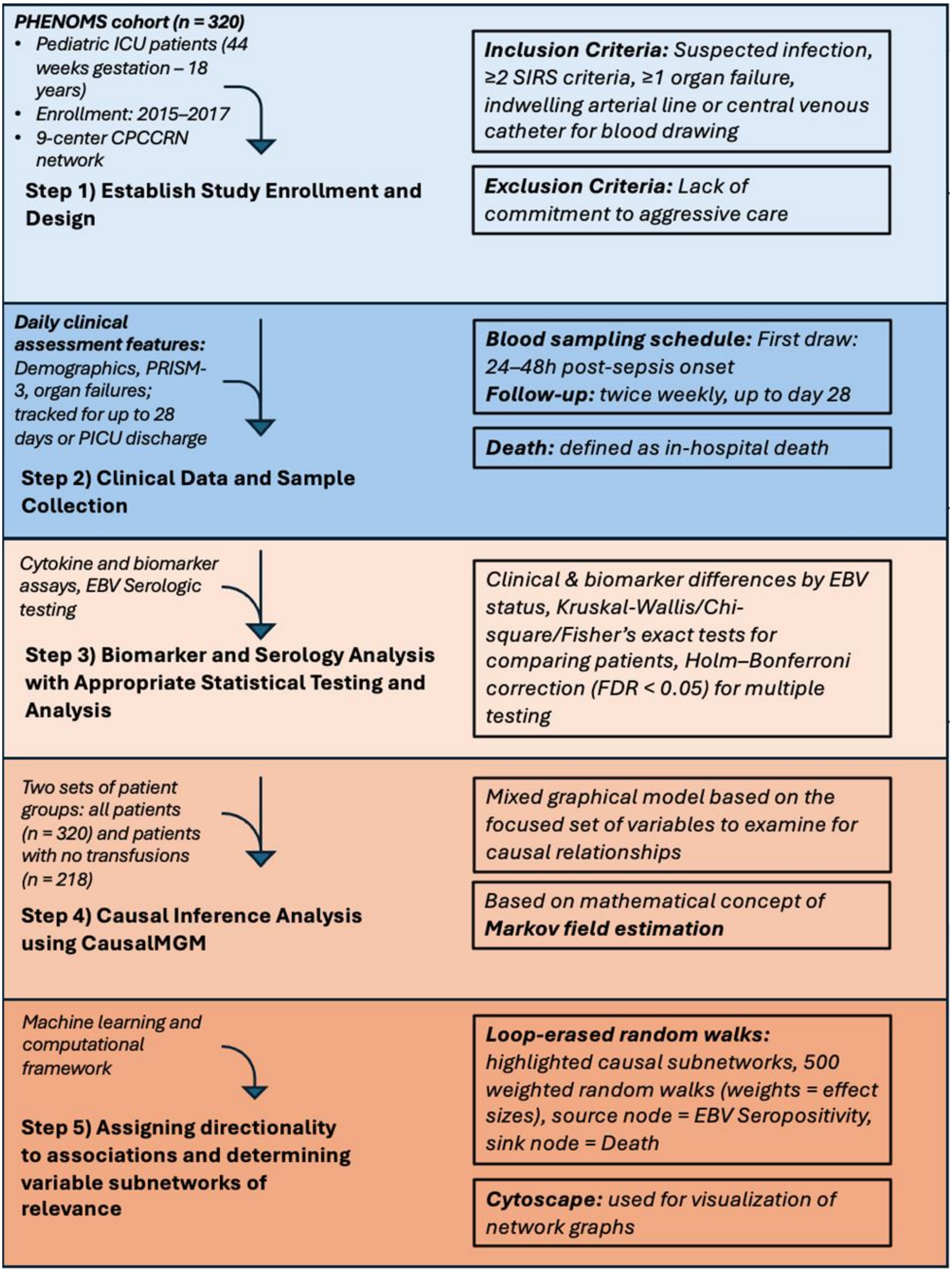

© 2025 Sriram A et al. *JAMA Network Open.*

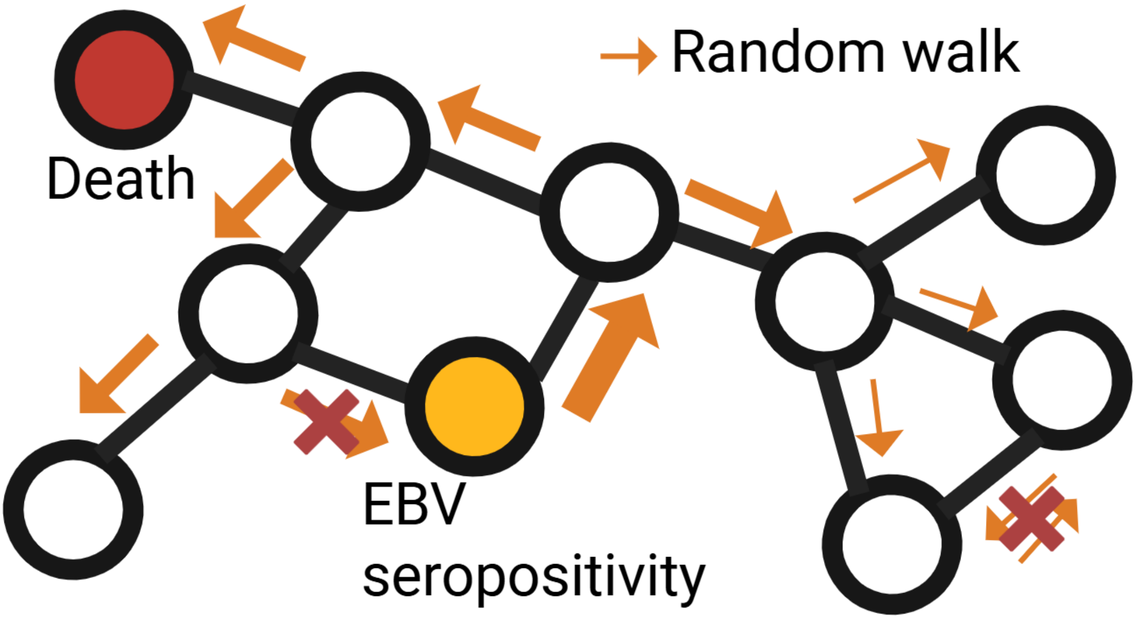

eFigure 2. Random Walk Illustration

© 2025 Sriram A et al. *JAMA Network Open.*

eMethods. Supplemental Extended DG Methods

DG learns the causal direction based on two fundamental assumptions, causal Markov condition and Faithfulness. Causal Markov condition states that each variable is conditionally independent of its non-descendants, given its direct parents in the true causal DAG. This is to enable the inference of conditional independence patterns in the data. The faithfulness assumption states that the observed (conditional) independences in the data correspond exactly to the independences in the underlying causal graph, making causal discovery unreliable. Like other causal learning methods based on the assumptions, our analyses would suffer with limited samples, violations of faithfulness, and latent confounders. With limited samples, statistical tests may yield spurious results or miss true dependencies, leading to incorrect edge inclusion/exclusion. With violations of faithfulness, near-unfaithfulness (e.g., small but non-zero effects) can lead to misinterpretation. With latent confounders, DG cannot distinguish between direct and spurious associations, potentially leading to incorrect edges."

© 2025 Sriram A et al. *JAMA Network Open.*

eTable 1. Biomarkers measured with serology using all patients and patients without transfusion

	All Patients (n = 320)		Patients without transfusion (n = 218)	
**Cytokines**	**Serology Positive**	**Serology Negative**	**Serology Positive**	**Serology Negative**
CRP	11.27 (5.02, 19.76)[a]	6.74 (2.45, 14.48)	11.75 (5.78, 18.83)[a]	6.19 (1.52, 14.49)
Ferritin	320.00 (126.60, 892.50)[a]	150.50 (76.20, 236.65)	187.00 (90.25, 474.08)[b]	141.10 (71.50, 226.20)
ADAMTS13	70.50 (56.75, 87.00)	74.00 (58.75, 92.25)	78.50 (58.25, 89.50)	74.00 (58.00, 91.00)
sFasLg	40.46 (25.16, 63.73)	51.42 (34.37, 84.91)[a]	45.34 (28.11, 68.74)	51.42 (34.37, 83.76)
Ex vivo TNF-α	416.43 (69.83, 1022.78)	459.27 (173.21, 972.82)	488.40 (179.85, 1048.55)	492.54 (168.81, 1049.22)
sCD163	316416.60 (199575.03, 523427.50)[b]	265931.40 (174016.02, 404024.22)	296578.90 (186109.57, 426677.73)	259400.00 (171420.65, 385922.63)
IFN-β	6.40 (6.40, 8.20)	6.40 (6.40, 6.65)	6.40 (6.40, 6.40)	6.40 (6.40, 7.60)
IL-22	26.55 (20.10, 34.50)	24.80 (20.10, 30.70)	24.80 (19.02, 33.90)	24.80 (20.10, 29.70)
IL-18	415.15 (286.60, 689.65)[c]	360.40 (216.80, 574.47)	383.90 (262.92, 603.45)	342.00 (206.48, 554.78)
IL-18BP	18113.70 (10659.20, 30923.45)[a]	13578.65 (6478.28, 21970.80)	16145.60 (9935.78, 29238.53) [b]	13039.65 (6243.37, 21075.90)
MIG/CXCL9	814.40 (476.63, 2468.50)[c]	677.10 (395.28, 1352.10)	689.60 (402.08, 2007.65)	668.80 (386.90, 1352.10)
IL-1β	2.80 (2.40, 3.20)	2.60 (2.10, 3.30)	2.80 (2.40, 3.20)	2.60 (2.10, 3.30)
IL-4	4.90 (3.50, 6.35)	5.10 (3.50, 7.00)	4.30 (3.50, 6.00)	5.10 (3.50, 7.00)
IL-6	9.70 (6.50, 23.25)[c]	7.65 (6.00, 13.20)	8.30 (5.80, 20.80)	7.50 (6.00, 12.98)
IL-8	61.10 (34.70, 154.70)[a]	46.10 (27.98, 69.80)	47.80 (32.02, 87.10)[c]	42.05 (26.40, 63.90)
IL-10	22.35 (17.80, 33.55)[c]	20.50 (16.15, 27.73)	21.70 (17.58, 32.67)	20.50 (15.62, 28.92)
IL-13	3.10 (3.10, 3.40)	3.10 (3.10, 5.00)	3.10 (3.10, 3.40)	3.10 (3.10, 4.47)
IL-17A	19.10 (16.50, 23.40)	18.30 (15.60, 21.70)	18.30 (15.82, 21.70)	17.40 (15.60, 21.70)
IFN-γ	2.80 (2.80, 3.00)	2.80 (2.80, 3.00)	2.80 (2.80, 2.80)	2.80 (2.80, 3.00)
IP-10/CXCL10	721.20 (330.42, 1555.25)	609.10 (321.85, 1901.55)	486.95 (298.78, 1575.35)	562.75 (315.88, 1706.83)

© 2025 Sriram A et al. *JAMA Network Open.*

MCP-1/CCL2	160.80 (77.35, 426.03)[b]	112.10 (53.85, 238.80)	119.30 (59.90, 277.88)	106.90 (51.78, 209.90)
MIP-1α	0.60 (0.60, 7.70)[c]	0.60 (0.60, 5.48)	0.60 (0.60, 4.68)	0.60 (0.60, 3.52)
MIP-1β	45.10 (31.88, 67.40)	42.95 (29.23, 64.22)	42.40 (30.70, 59.77)	41.75 (28.30, 61.57)
TNF-α	76.20 (58.40, 104.10)[c]	69.60 (52.30, 97.78)	66.90 (52.30, 98.02)	69.60 (49.20, 90.60)
MCP-3	92.40 (92.40, 166.00)	92.40 (92.40, 147.80)	119.50 (92.40, 161.45)	92.40 (92.40, 147.80)
IFN-α2	125.70 (105.80, 140.20)	125.70 (105.80, 144.40)	120.00 (105.80, 130.90)	125.70 (105.80, 144.40)
IL-1α	9.40 (9.40, 13.20)	9.40 (9.40, 14.00)	9.40 (9.40, 9.90)	9.40 (9.40, 13.20)
IL-2RA	378.80 (221.20, 584.03)	361.15 (238.50, 603.55)	300.70 (201.05, 448.12)	346.25 (222.98, 589.12)
IL-3	612.20 (529.00, 724.40)	636.60 (529.00, 749.40)	599.50 (504.33, 682.20)	636.60 (529.00, 734.60)
IL-16	557.35 (406.72, 777.05)	569.00 (426.22, 748.60)	530.25 (385.67, 697.45)	572.85 (426.70, 725.28)
M-CSF	33.10 (19.92, 63.78)[b]	24.30 (15.42, 43.20)	28.10 (15.08, 40.35)	22.00 (14.90, 37.28)
SCF	178.80 (116.80, 271.12)[b]	145.70 (116.45, 201.02)	133.85 (104.70, 200.35)	143.40 (113.42, 192.70)
TRAIL	35.40 (25.40, 50.40)	40.40 (30.40, 58.62)[c]	35.40 (25.70, 46.30)	40.40 (31.30, 63.00)[b]

Comparisons were performed between the EBV Serology positive and EBV Serology negative groups. KruskalWallis test was used for continuous variables. [a]: p-value < 0.001 [b]: p-value < 0.01 [c]: p-value < 0.05.

© 2025 Sriram A et al. *JAMA Network Open.*

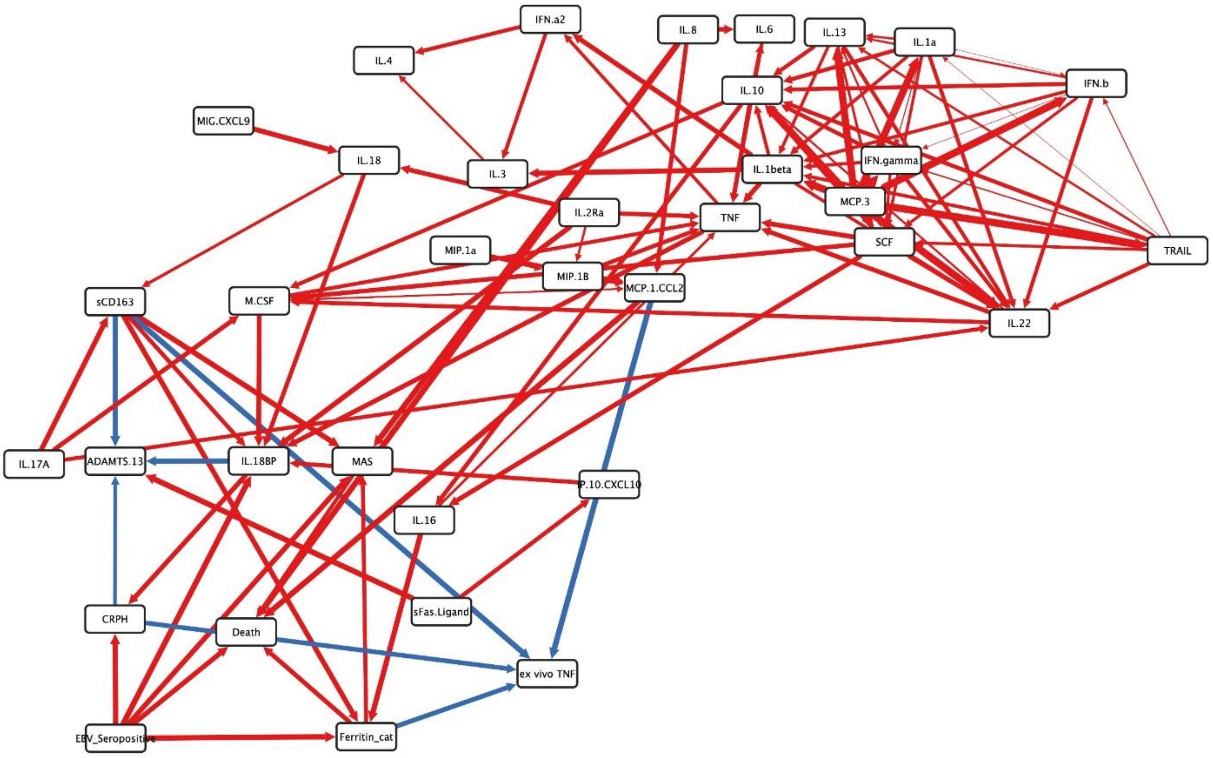

eFigure 3– Full Causal Association Network for EBV Seropositivity phenotype in All Patients (n = 320). Red arrows represent positive effect sizes, and blue arrows represent negative effect sizes. Arrow width is proportional to the magnitude of effect size.

© 2025 Sriram A et al. *JAMA Network Open.*

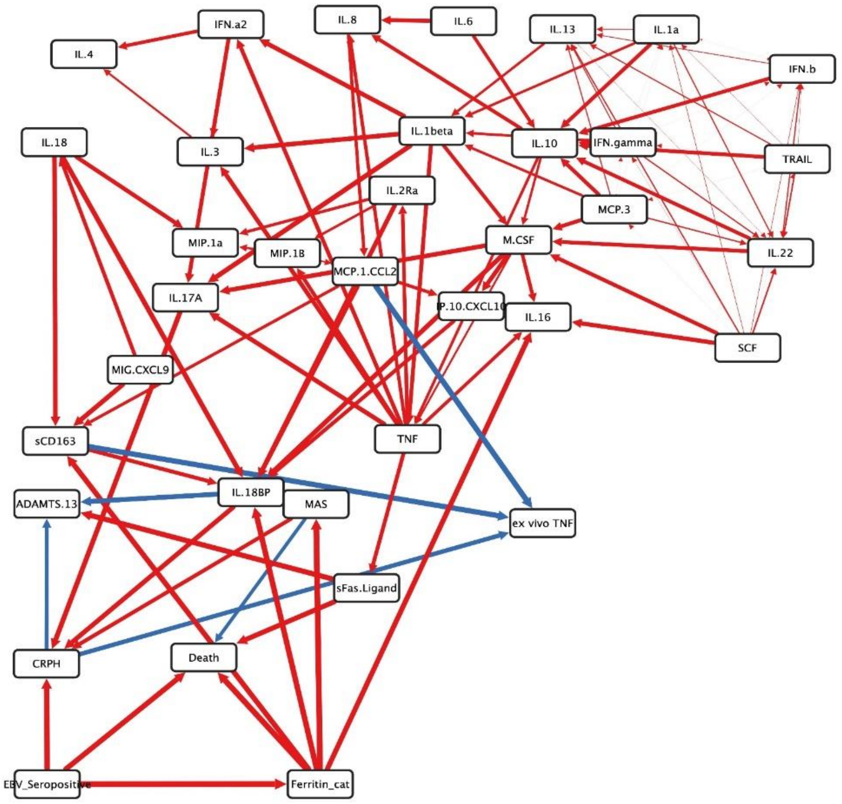

eFigure 4 – Full Causal Association Network for EBV Seropositivity phenotype in Patients without transfusion (N = 218). Red arrows represent positive effect sizes, and blue arrows represent negative effect sizes. Arrow width is proportional to the magnitude of effect size.

© 2025 Sriram A et al. *JAMA Network Open.*

eTable 2: Risk Ratios and E-values

E-value Method:

To assess any potential impact resulting from unmeasured confounding on observed causal associations between the exposures and the defined death phenotype, we ran an E-value analysis for each identified exposure-outcome association stratified by no transfusions compared to the whole population. Exposures included in the analysis were EBV Seropositivity, MAS, and ferritin (ferritin level coded as an integer with range 1-5; low = 1, high = 2, 3, 4, or 5). The E-value quantifies the minimum strength of association that an unmeasured confounder would need to have with both the exposure and the outcome, beyond the measured covariates, to fully explain away the observed risk ratio (RR) (The use of the E-value for sensitivity analysis, Chung et al. Journal of Clinical Epidemiology, Volume 163, 92 – 94, 2023). We used the formula proposed by VanderWeele and Ding (2017):

$$\boldsymbol{E} = \boldsymbol{RR} + \sqrt{\{\boldsymbol{RR} \cdot (\boldsymbol{RR} - \mathbf{1})\}}$$

where values closer to 1 indicate greater sensitivity to confounding, and larger E-values imply greater robustness. For each 2×2 exposure-outcome table, we applied a 0.5 continuity correction and computed the RR, 95% confidence interval (CI), and corresponding E-values. Calculations were performed using R software version 4.5.0.

E-value Result

E-value analysis showed that associations between the three exposures and death were differentially robust to unmeasured confounding by group status (non-transfused group and all patients, eTable 2).

EBV seropositivity in the full cohort had a risk ratio of 5.072 and E-value = 9.616, while the non-transfused patient group had a risk ratio of 7.46 and E-value of 14.39. Similarly, the association between ferritin and death exists across the full patient cohort (RR = 3.733, E-value = 6.927) consistent with the values in the non-transfused group (RR = 3.283, E-value = 6.02). In the full cohort, MAS had a RR of 2.804 with an E-value of 5.053, while in the non-transfused group, MAS had a RR of 1.395 and a lower E-value of 2.137. These results give merit to the idea that observed effects of EBV seropositivity and ferritin levels on death are more likely to reflect true associations and therefore are more reliable predictors of mortality across different patient groups in our data, while MAS may be more susceptible to unmeasured confounding.

eTable 2: Risk Ratios and E-values

Group	Exposure	Outcome	RR	CI	E_value	E_value_CI
All patients	EBV_Seropositivity	Death	5.072	1.913–13.447	9.616	3.235
Non-transfused	EBV_Seropositivity	Death	7.456	1.342–41.439	14.394	2.019
All patients	MAS	Death	2.804	1.237–6.354	5.053	1.779
Non-transfused	MAS	Death	1.395	0.089–21.924	2.137	22.029
All patients	Ferritin_high	Death	3.733	1.92–7.255	6.927	3.250
Non-transfused	Ferritin_high	Death	3.283	0.947–11.375	6.020	1.298

© 2025 Sriram A et al. *JAMA Network Open.*

© 2025 Sriram A et al. *JAMA Network Open.*

eTable 3 – Mediation analysis

Mediation Analysis METHODS

We evaluated whether ferritin levels and MAS acted as mediators of the EBV seropositivity and death relationship using the Baron-Kenny mediation analysis framework (MacKinnon DP, Fairchild AJ, Fritz MS. Mediation analysis. Annu Rev Psychol. 2007; 58:593-614. doi: 10.1146/annurev.psych.58.110405.085542. PMID: 16968208; PMCID: PMC2819368.). This approach involves sequentially fitting regression models to estimate the following pathways: (1) the total effect of the selected exposure (in this case, EBV seropositivity) on the outcome (death); (2) the effect of the exposure on the theorized mediator (in this case, ferritin and MAS); (3) the effect of the mediator on the outcome (in this case, outcome is death); and (4) the effect of the exposure on the outcome after adjusting for the mediator. A reduction in the exposure-outcome association after including the mediator, combined with a significant mediator-outcome effect, was taken as statistically driven evidence of mediation. Logistic regression was used for binary variables (e.g., death), and linear regression was used for continuous variables (e.g., ferritin). Mediation analysis was performed using R software version 4.5.0.

Mediation Analysis RESULTS

From the regression results as part of the mediation analysis, we observed that EBV seropositivity was associated with mortality (Estimate = 1.86, $p = 7.3\times10^{-4}$). When labeling ferritin as the mediator variable, EBV seropositivity was significantly associated with elevated ferritin levels (Estimate = 0.57, $p = 1.7\times10^{-7}$), and elevated ferritin was associated with mortality (Estimate = 0.63, $p = 1.3\times10^{-5}$). With both EBV seropositivity and ferritin included in the model, the effect of EBV seropositivity on death remained (Estimate = 1.52, $p = 7.4\times10^{-3}$), as did the ferritin effect (Estimate = 0.50, $p = 1.0\times10^{-3}$), indicating evidence of slight mediation. While EBV seropositivity was significantly associated with MAS (Estimate = 0.09, $p = 1.4\times10^{-3}$), and MAS was associated with death (Estimate = 1.17, $p = 0.034$), the association between MAS and death was no longer significant after adjusting for EBV seropositivity (Estimate = 0.76, $p = 0.18$). EBV seropositivity remained significantly associated with death even after adjustment (Estimate = 1.78, $p = 1.4\times10^{-3}$), suggesting that MAS may not play a mediating role in the EBV–death relationship. Alpha level for determining significance was set to 0.05 across all analyses.

© 2025 Sriram A et al. *JAMA Network Open.*

eTable 3: Mediation with Ferritin

Step	Estimate	Std.Error	p.value
EBV → Death	1.858	0.550	7.26e−04
EBV → Ferritin	0.572	0.107	1.71e−07
Ferritin → Death	0.630	0.145	1.31e−05
EBV → Death (adj)	1.516	0.566	7.39e−03
Ferritin → Death (adj)	0.498	0.152	1.01e−03

Death(adj): EBV adjusted for ferritin and Ferritin adjusted for EBV

---

eTable 3: Mediation with MAS

Step	Estimate	Std.Error	p.value
EBV → Death	1.858	0.550	0.000726
EBV → MAS	0.090	0.028	0.001410
MAS → Death	1.167	0.550	0.033900
EBV → Death (adj)	1.776	0.555	0.001370
MAS → Death (adj)	0.755	0.563	0.180000

Death(adj): EBV adjusted for MAS and MAS adjusted for EBV

© 2025 Sriram A et al. *JAMA Network Open.*

eTable 4 a-d – Structural Equation Modeling with and without mediation

eTables 4A and 4B - Normal Structural Equation Modeling Without Mediation

In the full cohort (n = 320), EBV seropositivity and ferritin were both independently associated with death. EBV seropositivity had a significant positive effect (standardized β = 0.143, *p* = 0.008), as did ferritin (β = 0.222, *p* < 0.001). MAS was not significantly associated with death (β = 0.045, *p* = 0.401).

In the non-transfused group (n = 218), EBV seropositivity remained significantly associated with death (β = 0.179, *p* = 0.007), while ferritin and MAS were not significant predictors.

eTables 4C and 4D - SEM With Mediation (Ferritin and MAS)

The mediation model for the full cohort showed partial mediation effect by ferritin. EBV seropositivity significantly predicted death (β = 0.072, *p* = 0.012) ferritin (β = 0.287, *p* < 0.001) and MAS (β = 0.178, *p* = 0.001), and ferritin was significantly associated with death (β = 0.219, *p* < 0.001). The indirect effect via ferritin was significant (β = 0.063, *p* = 0.001), but the path through MAS was not. Total effect of EBV on death was significant (β = 0.213, *p* < 0.001). However, the model fit was poor (RMSEA = 0.205, CFI = 0.827).

In the non-transfused group, no significant mediation was detected. EBV showed a stronger direct effect on death (β = 0.178, *p* = 0.008). Relationships via paths through ferritin and MAS were non-significant.

All structural equation modeling was done with the lavaan R package.
Rosseel Y (2012). "lavaan: An R Package for Structural Equation Modeling." *Journal of Statistical Software*, **48**(2), 1–36. doi:10.18637/jss.v048.i02.

eTable 4A. SEM Without Mediation — Full Cohort (n = 320)

Predictor	Estimate	Std. Error	z-value	p-value	Std. Est.
EBV_Seropositivity → Death	0.083	0.031	2.661	0.008	0.143
MAS → Death	0.052	0.061	0.841	0.401	0.045
Ferritin_cat → Death	0.064	0.016	4.117	<0.001	0.222
R^2 (Death)	–	–	–	–	0.091
RMSEA	–	–	–	–	0.227
CFI	–	–	–	–	0.370

eTable 4B. SEM Without Mediation — Non-Transfused (n = 218)

Predictor	Estimate	Std. Error	z-value	p-value	Std. Est.
EBV_Seropositivity → Death	0.072	0.027	2.696	0.007	0.179
MAS → Death	-0.057	0.075	-0.769	0.442	-0.051
Ferritin_cat → Death	0.024	0.018	1.318	0.187	0.087
R^2 (Death)	–	–	–	–	0.047

© 2025 Sriram A et al. *JAMA Network Open.*

RMSEA	–	–	–	–	0.091
CFI	–	–	–	–	0.575

eTable 4C. Mediation SEM — Full Cohort (n = 320)

Pathway	Estimate	Std. Error	z-value	p-value	Std. Est.
EBV → Death	0.083	0.033	2.511	0.012	0.142
EBV → Ferritin	0.572	0.107	5.364	<0.001	0.287
Ferritin → Death	0.064	0.016	3.943	<0.001	0.219
EBV → MAS	0.090	0.028	3.231	0.001	0.178
MAS → Death	0.052	0.062	0.827	0.408	0.045
Indirect via Ferritin	0.037	0.012	3.177	0.001	0.063
Indirect via MAS	0.005	0.006	0.801	0.423	0.008
Total Indirect	0.041	0.013	3.200	0.001	0.071
Total Effect	0.124	0.032	3.895	<0.001	0.213
R^2 (Death)	–	–	–	–	0.091
RMSEA	–	–	–	–	0.205
CFI	–	–	–	–	0.827

eTable 4D. Mediation SEM — Non-Transfused (n = 218)

Pathway	Estimate	Std. Error	z-value	p-value	Std. Est.
EBV → Death	0.072	0.027	2.646	0.008	0.178
EBV → Ferritin	0.275	0.099	2.776	0.006	0.185
Ferritin → Death	0.024	0.018	1.296	0.195	0.087
EBV → MAS	0.018	0.024	0.763	0.446	0.052
MAS → Death	-0.057	0.075	-0.768	0.442	-0.051
Indirect via Ferritin	0.006	0.006	1.174	0.240	0.016
Indirect via MAS	-0.001	0.002	-0.541	0.588	-0.003
Total Indirect	0.005	0.006	0.927	0.354	0.013
Total Effect	0.077	0.027	2.885	0.004	0.192
R^2 (Death)	–	–	–	–	0.047
RMSEA	–	–	–	–	0.000
CFI	–	–	–	–	1.000

© 2025 Sriram A et al. *JAMA Network Open.*

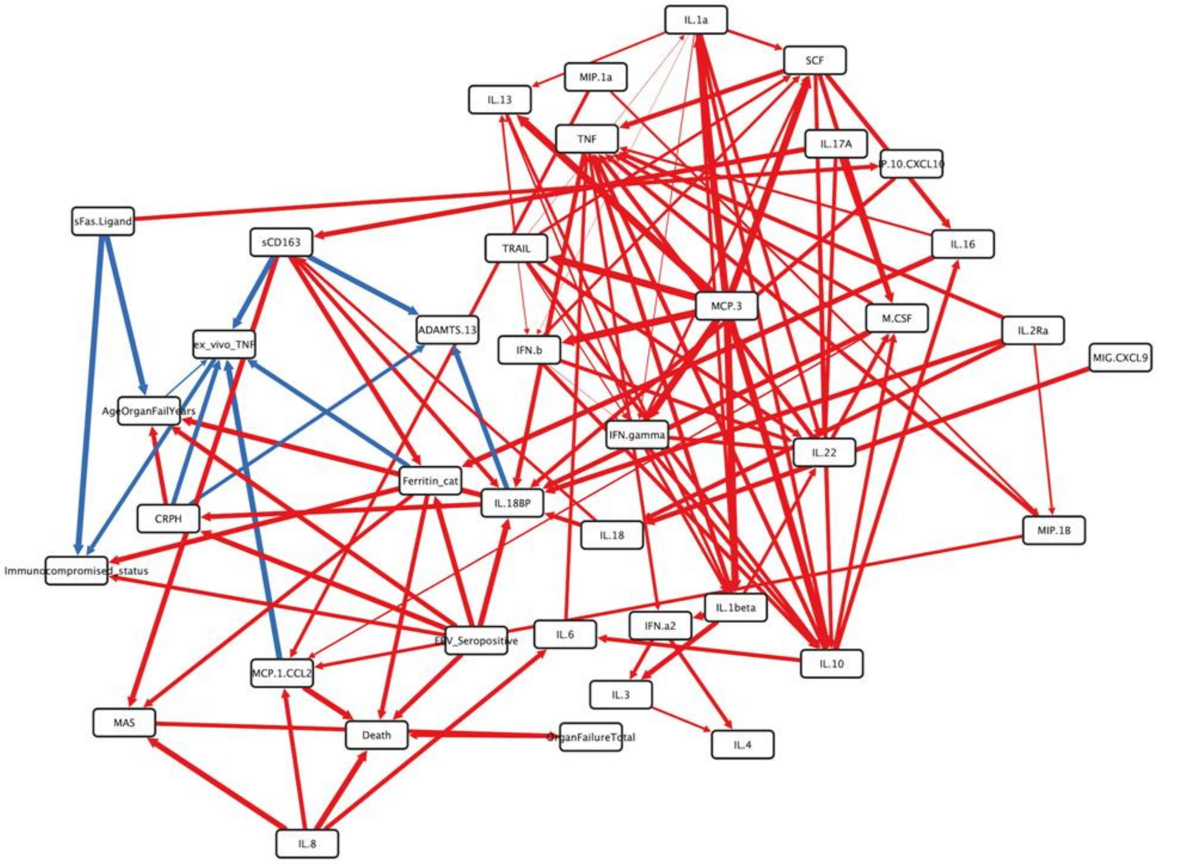

eFigure 5. Full Causal Association Network for EBV Seropositivity phenotype in all patients (n = 320) including admission confounders increased age, organ failures, severity of illness (PRISM score), and immunocompromise as nodes, . Red arrows represent positive effect sizes, and blue arrows represent negative effect sizes. Arrow width is proportional to the magnitude of effect size.

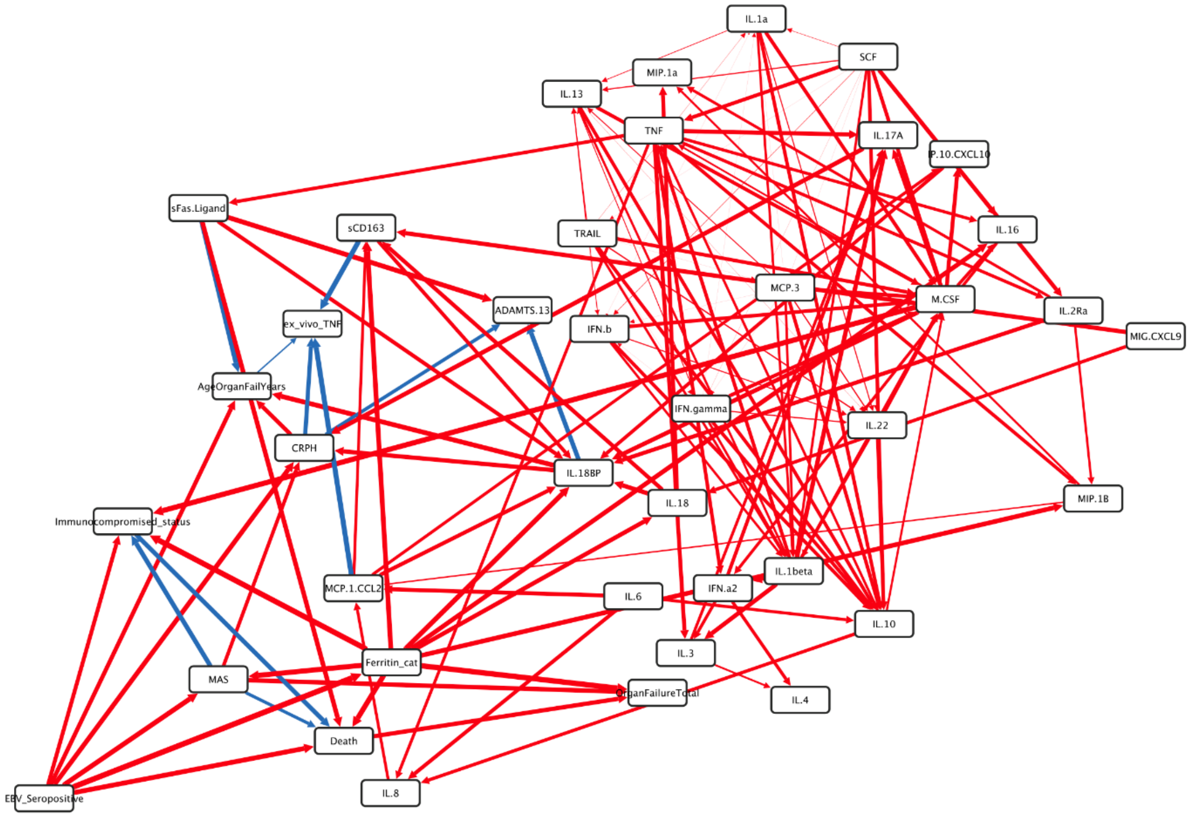

eFigure 6.

Full Causal Association Network for EBV Seropositivity phenotype in patients without transfusion (n = 218) including admission confounders increased age, organ failures, severity of illness (PRISM score), and immunocompromise as nodes. Red arrows represent positive effect sizes, and blue arrows represent negative effect sizes. Arrow width is proportional to the magnitude of effect size.

© 2025 Sriram A et al. *JAMA Network Open.*

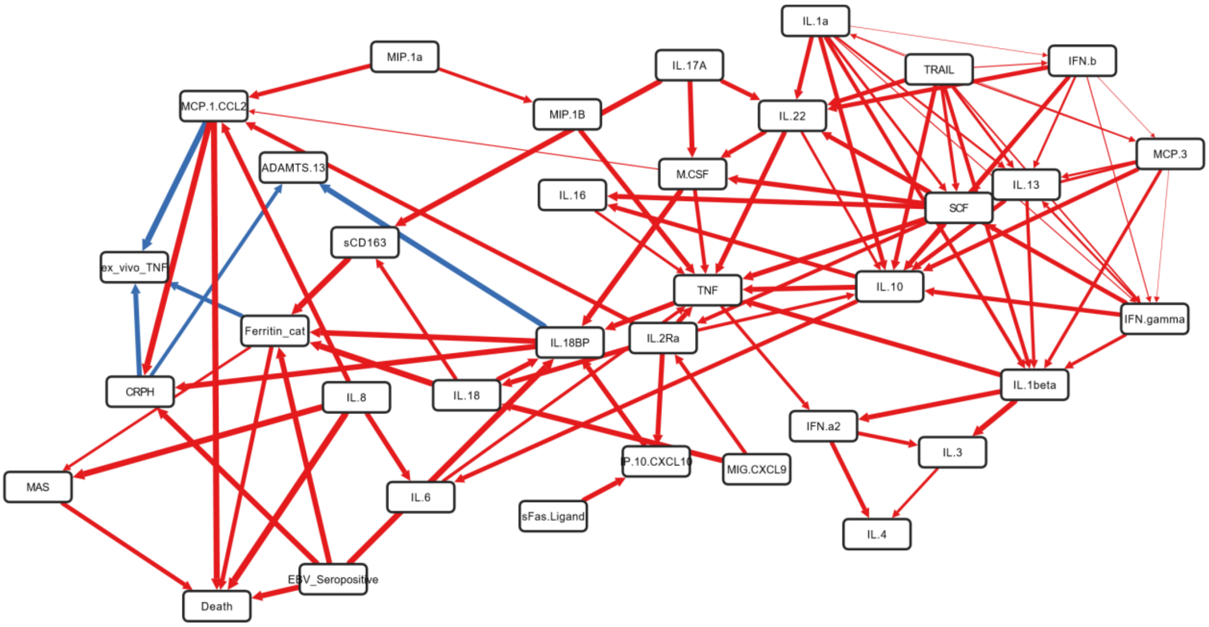

eFigure 7 – Full Causal Association Network for EBV Seropositivity phenotype in all patients greater than 18 months (n = 237). Red arrows represent positive effect sizes, and blue arrows represent negative effect sizes. Arrow width is proportional to the magnitude of effect size.

© 2025 Sriram A et al. *JAMA Network Open.*

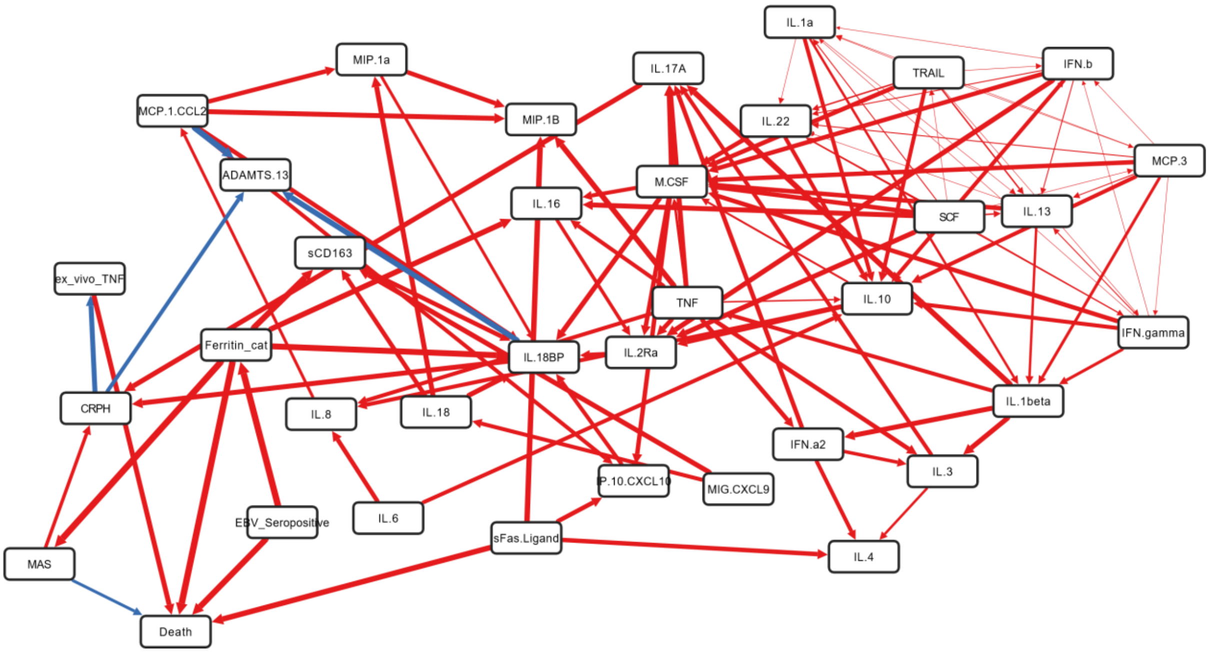

eFigure 8 – Full Causal Association Network for EBV Seropositivity phenotype in all patients greater than 18 months without transfusion (N = 167). Red arrows represent positive effect sizes, and blue arrows represent negative effect sizes. Arrow width is proportional to the magnitude of effect size.

© 2025 Sriram A et al. *JAMA Network Open.*

eTable 5 Outcome characteristics according to EBV Seropositivity phenotype in all patients and in patients without transfusion.

	All Patients (N = 320)		Patients without transfusion (N = 218)	
**Outcome**	**Serology Positive**	**Serology Negative**	**Serology Positive**	**Serology Negative**
No. of patients, N (%)	172 (53.75)	148 (46.25)	95 (43.58)	123 (56.42)
Lymphopenia, N (%)	96 (55.81)	75 (50.68)	43 (45.26)	59 (47.97)
MechVent, N (%)	146 (84.88)	127 (85.81)	81 (85.26)	103 (83.74)
ECMO, N (%)	10 (5.81) [b]	0 (0.00)	0 (0.00)	0 (0.00)
CRRT, N (%)	12 (6.98) [c]	2 (1.35)	3 (3.16)	1 (0.81)
PLEX, N (%)	3 (1.74)	0 (0.00)	1 (1.05)	0 (0.00)
Mortality, N (%)	26 (15.12) [a]	4 (2.70)	8 (8.42) [c]	1 (0.81)
IPMOF, N (%)	46 (26.74) [b]	17 (11.49)	20 (21.05)	14 (11.38)
TAMOF, N (%)	22 (12.79) [a]	2 (1.35)	4 (4.21)	1 (0.81)
SMOF, N (%)	4 (2.33)	0 (0.00)	1 (1.05)	0 (0.00)
MAS, N (%)	12 (6.98) c	2 (1.35)	3 (3.16)	1 (0.81)

Abbreviations: IQR, interquartile range; MechVent, Mechanical Ventilation; ECMO, Extracorporeal Membrane Oxygenation; CRRT, Continuous Renal Replacement Therapies; PLEX, Plasma Exchange; IPMOF, immunoparalysis associated multiple organ failure; TAMOF, thrombocytopenia associated multiple organ failure; MAS, macrophage activation syndrome. Lymphopenia = Absolute Lymphoycte Count < 1,000/mm^3

Comparisons were performed between the group with EBV latency and no EBV, and the group with EBV Serology positive and EBV Serology negative. The $\chi 2$ test or the fisher's exact test (group sample size < 10) was used for discrete variables. [a]: p-value < 0.001 [b]: p-value < 0.01 [c]: p-value < 0.05.

© 2025 Sriram A et al. *JAMA Network Open.*

eTable 6. Admission Characteristics of EBV latency without presumed reactivation compared to no EBV infection using all patients and patients without transfusion

	All Patients (n = 280)		Patients without transfusion (n = 191)	
**Admission Characteristic**	**EBV Latent**	**No EBV**	**EBV Latent**	**No EBV**
No. of patients, N (%)	139 (49.64)	141 (50.36)	73 (38.22)	118 (61.78)
Age, median (IQR), y	7 (2, 13)[b]	3 (1, 9)	8 (3, 13)[a]	3 (1, 10)
Sex, N (%)				
Female	67 (48.20)	62 (43.97)	37 (50.68)	53 (44.92)
Male	72 (51.80)	79 (56.03)	36 (49.32)	65 (55.08)
Race, N (%)				
White	93 (66.91)	103 (73.05)	52 (71.23)	86 (72.88)
Black	30 (21.58)	22 (15.60)	15 (20.55)	18 (15.25)
Asian	5 (3.60)	7 (4.96)	2 (2.74)	6 (5.08)
Unknown	11 (7.91)	9 (6.38)	4 (5.48)	8 (6.78)
Ethnicity, N (%)				
Non-Hispanic	112 (80.58)	114 (80.85)	58 (79.45)	91 (77.12)
Hispanic	21 (15.11)	24 (17.02)	14 (19.18)	24 (20.34)
Unknown	6 (4.32)	3 (2.13)	1 (1.37)	3 (2.54)
Previous healthy, N (%)	57 (41.01)	76 (53.90)[c]	35 (47.95)	68 (57.63)
Immunocompromised, N (%)	49 (35.25)[a]	14 (9.93)	15 (20.55)[c]	10 (8.47)
PRISM Score, median (IQR)	9 (4.5, 15)[c]	7 (3, 12)	7 (3, 12)	6.5 (3, 11)
OFI, median (IQR)	2 (1, 2)[b]	1 (1, 2)	1 (1, 2)	1 (1, 2)
Bacterial infection, N (%)	53 (38.13)	43 (30.50)	24 (32.88)	33 (27.97)
Viral infection, N (%)	27 (19.42)	55 (39.01)[a]	15 (20.55)	47 (39.83)[a]
Co-infection, N (%)	10 (7.19)	10 (7.09)	4 (5.48)	6 (5.08)
Fungal infection, N (%)	1 (0.72)	0 (0)	0 (0)	0 (0)

Abbreviations: IQR, interquartile range; PRISM, Pediatric Risk of Mortality Index; OFI, organ failure index, is an integer score reflecting the number of organ failures. OFI Scores are either 0 or 1 for cardiovascular, hepatic, hematologic, respiratory, neurological, and renal, and summed for total range of 0 to 6. Co-infection = bacteria + virus infections. Immunocompromise – Cancer, transplantation, use of immune suppressant therapies;

Comparisons were performed between the group with EBV latency and no EBV, and the group with EBV Serology positive and EBV Serology negative. Kruskal-Wallis test was used for continuous variables, the $\chi 2$ test or the fisher's exact test (group sample size < 10) was used for discrete variables. [a]: p-value < 0.001 [b]: p-value < 0.01[c]: p-value < 0.05.

© 2025 Sriram A et al. *JAMA Network Open.*

eTable 7 Biomarkers measured EBV latency without presumed reactivation compared to no EBV infection using all patients and patients without transfusion

	All Patients (n = 280)		Patients without transfusion (n = 191)	
**Cytokines**	**EBV latent**	**No EBV**	**EBV latent**	**No EBV**
CRP	10.79 (4.70, 18.44)[b]	6.32 (2.38, 14.34)	11.35 (5.25, 17.06)[b]	6.11 (1.46, 14.34)
Ferritin	331.20 (128.05, 984.50)[a]	153.00 (77.20, 235.00)	181.60 (88.00, 411.60)[c]	146.00 (74.60, 225.52)
ADAMTS13	71.00 (57.00, 87.50)	74.00 (59.00, 92.00)	81.00 (59.00, 93.00)	74.50 (58.25, 91.00)
sFasLg	40.76 (24.27, 63.81)	50.79 (34.43, 84.39)[a]	43.22 (26.25, 63.99)	50.62 (34.43, 81.95)
Ex vivo TNF-α	439.79 (70.93, 1031.60)	463.29 (209.46, 1049.22)	498.76 (217.04, 1046.53)	494.92 (167.49, 1049.22)
sCD163	315178.00 (192552.75, 525649.50)[c]	263870.90 (174099.30, 407903.60)	296249.40 (177668.90, 430278.70)	252867.35 (172126.02, 387224.07)
IFN-β	6.40 (6.40, 8.20)	6.40 (6.40, 6.40)	6.40 (6.40, 6.40)	6.40 (6.40, 7.15)
IL-22	27.10 (20.10, 34.80)	24.80 (19.40, 30.70)	26.00 (18.90, 34.20)	24.80 (20.10, 29.50)
IL-18	415.90 (273.00, 695.40)[c]	359.60 (216.80, 566.40)	383.10 (254.40, 608.10)	337.25 (205.62, 547.80)
IL-18BP	17695.00 (10627.70, 30468.70)[a]	13489.40 (6457.80, 21913.20)	15422.60 (10087.80, 28399.10)[c]	12787.90 (6241.33, 20442.92)
MIG/CXCL9	810.50 (473.65, 2474.60)[c]	675.00 (387.30, 1333.90)	700.00 (401.10, 2013.60)	662.60 (379.92, 1307.98)
IL-1β	2.80 (2.10, 3.20)	2.60 (2.10, 3.30)	2.80 (2.10, 3.20)	2.60 (2.10, 3.27)
IL-4	5.10 (3.50, 6.50)	5.10 (3.50, 7.00)	4.30 (3.50, 6.00)	5.10 (3.50, 6.95)
IL-6	9.10 (6.35, 21.45)	7.80 (6.00, 13.20)	7.30 (5.80, 20.90)	7.50 (6.00, 11.28)
IL-8	60.70 (35.50, 152.00)[a]	46.10 (27.60, 69.60)	46.90 (31.90, 87.90)[c]	41.20 (26.40, 60.70)
IL-10	23.40 (17.80, 36.70)[b]	20.50 (16.30, 27.20)	22.20 (18.10, 33.40)	20.20 (15.48, 27.42)
IL-13	3.10 (3.10, 3.40)	3.10 (3.10, 5.00)	3.10 (3.10, 3.40)	3.10 (3.10, 4.82)
IL-17A	19.10 (16.50, 22.60)	18.30 (15.60, 21.70)	17.40 (15.60, 21.70)	17.85 (15.60, 21.70)
IFN-γ	2.80 (2.80, 3.00)	2.80 (2.80, 3.00)	2.80 (2.80, 2.80)	2.80 (2.80, 3.00)
IP-10/CXCL10	658.50 (308.20, 1483.10)	608.40 (319.30, 1895.10)	443.90 (286.90, 1538.60)	561.50 (311.83, 1663.53)
MCP-1/CCL2	159.30 (77.20, 402.65)[b]	111.70 (52.80, 229.90)	115.80 (59.70, 274.80)	106.45 (51.12, 200.30)
MIP-1α	0.60 (0.60, 8.85)[c]	0.60 (0.60, 5.40)	0.60 (0.60, 4.60)	0.60 (0.60, 2.92)
MIP-1β	44.50 (31.75, 69.25)	42.80 (29.30, 64.60)	42.40 (31.00, 66.10)	41.45 (28.30, 61.93)
TNF-α	74.90 (58.40, 104.10)	69.60 (52.30, 97.20)	66.90 (55.10, 98.30)	69.60 (48.45, 88.90)
MCP-3	92.40 (92.40, 166.00)	92.40 (92.40, 147.80)	92.40 (92.40, 147.80)	92.40 (92.40, 147.80)
IFN-α2	125.70 (105.80, 140.20)	125.70 (105.80, 144.40)	120.00 (105.80, 135.70)	125.70 (105.80, 144.40)
IL-1α	9.40 (9.40, 14.80)	9.40 (9.40, 16.40)	9.40 (9.40, 9.90)	9.40 (9.40, 13.20)

© 2025 Sriram A et al. *JAMA Network Open.*

IL-2RA	400.00 (221.20, 585.70)	368.20 (239.40, 597.50)	300.70 (199.20, 449.00)	347.00 (224.80, 577.95)
IL-3	612.20 (529.00, 724.40)	636.60 (529.00, 764.10)	599.50 (496.10, 682.20)	636.60 (529.00, 734.60)
IL-16	569.80 (410.25, 787.00)	562.60 (424.80, 743.30)	525.40 (378.20, 689.20)	565.40 (426.70, 717.90)
M-CSF	33.70 (20.15, 68.00)[a]	24.30 (15.60, 44.10)	30.00 (14.90, 47.40)	22.00 (14.90, 39.42)
SCF	188.80 (118.80, 272.10)[b]	145.70 (116.80, 200.70)	142.60 (106.00, 223.20)	142.75 (112.08, 192.20)
TRAIL	36.60 (25.40, 52.30)	40.40 (30.40, 58.00)	36.60 (25.40, 47.90)	40.40 (31.93, 62.38)[c]

Comparisons were performed between the group with EBV latency and no EBV, and the group with EBV Serology positive and EBV Serology negative. Kruskal-Wallis test was used for continuous variables. [a]: p-value < 0.001 [b]: p-value < 0.01 [c]: p-value < 0.05.

© 2025 Sriram A et al. *JAMA Network Open.*

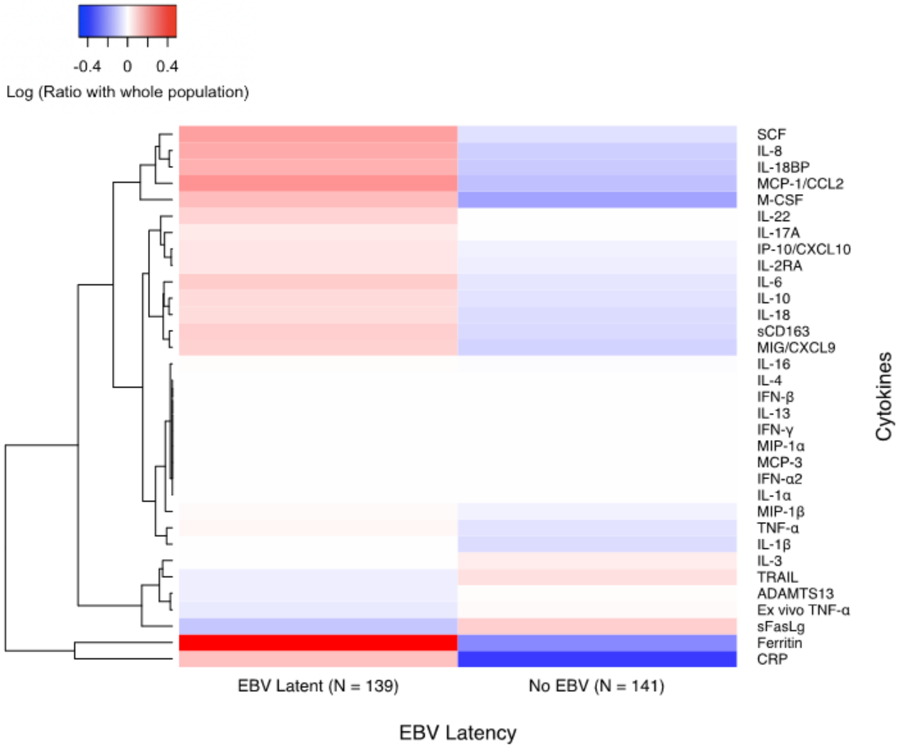

eFigure 9. Cytokine Heatmap of EBV latent without presumed reactivation (EBV VCA + / EBV PCR -) and no EBV (EBV VCA - / EBV PCR -) groups (n = 280). The heatmap shows the log ratio of the median biomarker values for various markers of the host response and their hierarchical cluster relationships. Red represents a greater median biomarker value for that group compared with the median for the entire study cohort, whereas blue represents a lower median biomarker value compared with the median for the entire study cohort.

© 2025 Sriram A et al. *JAMA Network Open.*

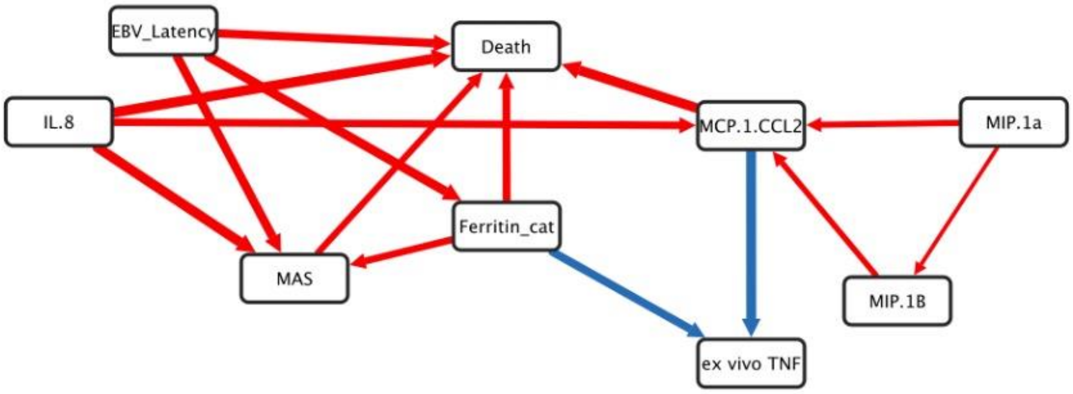

eFigure 10. Abridged Causal Association Network for EBV Latency without presumed reactivation compared to No EBV infection in all patients (n = 280). Red arrows represent positive effect sizes, and blue arrows represent negative effect sizes. Arrow width is proportional to the magnitude of effect size.

© 2025 Sriram A et al. *JAMA Network Open.*

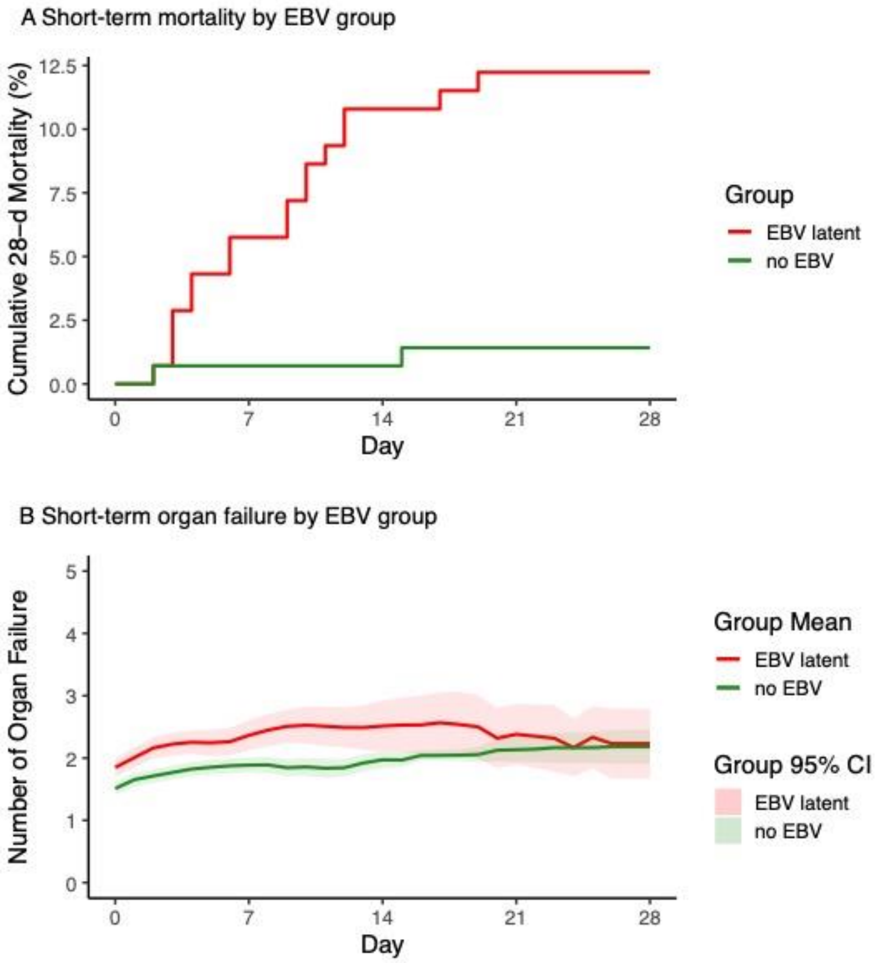

eFigure 11. **Outcome curves over 28 days among all patients with EBV latency without presumed reactivation and all patients without EBV infection.** A. Short-term mortality of all patients (n = 280, p = 0.00051) B. Short-term organ failure of all patients (n = 280, p = 0.18). The mean numbers of organ failures and 95% confidence intervals (CI) are calculated each day by non-nested observation, where we do not carry forward the OFI at the time the patient leaves the PICU alive or dead.

© 2025 Sriram A et al. *JAMA Network Open.*

eTable 8, Outcomes according to EBV latency using all patients and patients without transfusion

	All Patients (N = 280)		Patients without transfusion (N = 191)	
**Outcome**	**EBV latent**	**No EBV**	**EBV latent**	**No EBV**
No. of patients, N (%)	139 (49.64)	141 (50.36)	73 (38.22)	118 (61.78)
Lymphopenia, N (%)	82 (58.99)	71 (50.35)	28 (38.36)	57 (48.31)
MechVent, N (%)	115 (82.73)	120 (85.11)	60 (82.19)	98 (83.05)
ECMO, N (%)	8 (5.76)[b]	0 (0.00)	0 (0.00)	0 (0.00)
CRRT, N (%)	11 (7.91) c	2 (1.42)	3 (4.11)	1 (0.85)
PLEX, N (%)	2 (1.44)	0 (0.00)	1 (1.37)	0 (0.00)
Mortality, N (%)	21 (15.11) [a]	4 (2.84)	5 (6.85) c	1 (0.85)
IPMOF, N (%)	30 (21.58) [c]	14 (9.93)	11 (15.07)	12 (9.93)
TAMOF, N (%)	16 (11.51) [a]	2 (1.42)	3 (4.11)	1 (0.85)
SMOF, N (%)	2 (1.44)	0 (0.00)	0 (0.00)	0 (0.00)
MAS, N (%)	10 (7.19) [b]	1 (0.71)	1 (1.37)	1 (0.85)

Abbreviations: IQR, interquartile range; MechVent, Mechanical Ventilation; ECMO, Extracorporeal Membrane Oxygenation; CRRT, Continuous Renal Replacement Therapies; PLEX, Plasma Exchange; IPMOF, immunoparalysis associated multiple organ failure; TAMOF, thrombocytopenia associated multiple organ failure; MAS, macrophage activation syndrome. Lymphopenia = Absolute Lymphocyte Count < 1,000/mm^3

Comparisons were performed between the group with EBV latency and no EBV, and the group with EBV Serology positive and EBV Serology negative. The $\chi 2$ test or the fisher's exact test (group sample size < 10) was used for discrete variables. [a]:p-value < 0.001 [b]:p-value < 0.01 [c]p-value < 0.05.

© 2025 Sriram A et al. *JAMA Network Open.*
