# Supplementary material for: Epstein-Barr Virus Seropositivity, Immune Dysregulation, and Mortality in Pediatric Sepsis
Source: JAMA Netw Open. 2025 Aug 19;8(8):e2527487. doi: 10.1001/jamanetworkopen.2025.27487 (PMC12365707; doi:10.1001/jamanetworkopen.2025.27487)
Supplement: Supplement 2. — Data Sharing Statement [file jamanetwopen-e2527487-s002.pdf]

Data Sharing Statement

Sriram. Epstein-Barr Virus Seropositivity, Immune Dysregulation, and Mortality in Pediatric Sepsis. *JAMA Netw Open*. Published August 19, 2025.
doi:10.1001/jamanetworkopen.2025.27487

Data
Data available: Yes
Data types: Deidentified participant data, Data dictionary
How to access data: The data will be available at the NICHD DASH website.
When available: With publication

Supporting Documents
Document types: None

Additional Information
Who can access the data: Available only to researchers whose proposed use of the data is approved by the NICHD-CPCCRN DCC IRB
Types of analyses: Research analysis only. Not for commercialization puposes.
Mechanisms of data availability: Without investigator support. After approval of proposal by NICHD-CPCCRN designated IRB.
Any additional restrictions: Not for commercial use
